# Supplementary material for: Development of a tool to assess beliefs about mythical causes of cancer: the Cancer Awareness Measure Mythical Causes Scale
Source: BMJ Open. 2018 Dec 14;8(12):e022825. doi: 10.1136/bmjopen-2018-022825 (PMC6303629; doi:10.1136/bmjopen-2018-022825)
Supplement: Supplementary file 1 [file bmjopen-2018-022825supp001.pdf]

Supplementary Table 1. Citation generated from search terms, May 2015

Step	Term Category	Search string	Medline/ EMBASE	PsycINFO/ EXTRA	All together-deduplicated
1	"Cancer" terms^	(cancer OR neoplasm).me	2,776,856	47,990	
2	"Belief" terms^	(caus* or attribute* or belie* or perce* or (illness adj representation*) or know* or aware* or thought* or idea*).me	697,570	1,386,598	
3	"Wrong" terms	(false* or incorrect* or myth* or erroneous* or wrong* or mistaken* or error or untrue or fallacious).mp	921,613	125,798	
4	"Cancer patient" terms	cancer adj patient*.tw	302,437	11,606	
5	Combinatio n	1 AND 2 AND 3	784	534	
6	Exclusion	5 NOT 4	716	460	
7	Limits	Human, English	632	417	**999**

^Mesh terms/Subject heading terms for Medline/Embase only (otherwise .tw)
