# Supplementary material for: Development of a tool to assess beliefs about mythical causes of cancer: the Cancer Awareness Measure Mythical Causes Scale
Source: BMJ Open. 2018 Dec 14;8(12):e022825. doi: 10.1136/bmjopen-2018-022825 (PMC6303629; doi:10.1136/bmjopen-2018-022825)
Supplement: Supplementary file 2 [file bmjopen-2018-022825supp002.pdf]

Supplementary information

Appendix A: Topic guide

SEMI-STRUCTURED INTERVIEW SCHEDULE (CANCER AWARENESS)

**Objective**<br>In-depth exploration of the incorrect causal beliefs about cancer in the UK general population sample and identifying existing socioeconomic inequalities.<br><br>To generate items to develop a valid and reliable tool for assessing cancer awareness in the public.

Beginning the interview

- Introduction of researcher; topics to be covered/structure of interview; confidentiality; timing; confirming consent for tape-recording

1.1 Background

- Name RP, a masters' student in Health Psychology at UCL
- Interested in understanding and knowing about people's understanding of cancer. Don't worry there are no right or wrong answers. We are just interested in your ideas.

1.2 Interview Outline

- If that's ok, I will ask you a couple of questions about your views on cancer in general,
- Also interested to hear about your beliefs about the different causes of cancer
- At the end will describe a new cancer awareness assessment tool we are developing at UCL and I would very much appreciate to hear your views about that tool

1.3 Interview Arrangements

- Should take around half an hour, but we might find lots to talk about, so please go into as much detail as possible.
- Ok to record interview (consent)
- Of course it's all completely anonymous/confidential and recordings will be transcribed

During the interview (main themes to be covered)

2.1. Questions exploring the causal beliefs or risk perceptions of cancer:

2.1.1. How much do you understand about cancer? Have you have had any experience with cancer through friends or relatives?

2.1.2. A number of different things have been linked to the development of cancer, but new discoveries are often being made. What do you think are the most common causes of cancer?

2.1.3. What things do you think affects a person’s chances of developing cancer?

2.1.4. What are the best ways to avoid getting cancer?

2.1.5. Do you think that certain behaviours or risk factors are more important for specific people (e.g. prompt: men vs. women, younger vs. older; different ethnic groups)?

2.1.6 We have been asking all our interviewees this question, what do you think our chances of developing cancer is in your lifetime? Would you say it is higher or lower compared to an average person?

<u>Prompts for questions:</u>

- “What else?”
- “Take your time to think about it”

3. Ending the interview

- Anything else participants would like to say?
- Any important issues not raised?
- Any other questions regarding research?
- Debriefing about study
- Provision of researcher (RP) and principal researcher’s (LS/SS) contact information, should anything else come up
- Thanks
