# Supplementary material for: Development of a tool to assess beliefs about mythical causes of cancer: the Cancer Awareness Measure Mythical Causes Scale
Source: BMJ Open. 2018 Dec 14;8(12):e022825. doi: 10.1136/bmjopen-2018-022825 (PMC6303629; doi:10.1136/bmjopen-2018-022825)
Supplement: Supplementary file 3 [file bmjopen-2018-022825supp003.pdf]

LONDON'S GLOBAL UNIVERSITY

UCL

Cancer Information Leaflet

Known risk factors and common myths

IBAC Study Educational Leaflet

By: Emma Fox

University College London, Gower Street, London WC1E 6BT
Tel: +44 (0)20 7679 2000
email@ucl.ac.uk
www.ucl.ac.uk

What is cancer?

Cancer is the name of a collection of diseases where the cells of the body begin to divide abnormally. These cells can become invasive, and grow out of control, spreading into surrounding tissues. Many cancers grow to form solid masses of tissue, known as tumours.

Cancer can start almost anywhere in the human body, and there are more than 100 different types of cancer.

What causes cancer?

Changes to genes, called mutations, play a vital role in the development of cancer. Some of the gene changes that lead to cancer may be inherited. Most gene mutations, however, happen after birth.

It is not usually possible to say exactly how a person develops cancer. Nevertheless, research has shown that certain risk factors may increase the likelihood of an individual developing cancer. These include factors such as exposure to ultraviolet rays found in sunlight, as well as lifestyle behaviours such as smoking and heavy alcohol consumption. Other risk factors include family history and age.

Myths and misconceptions about cancer

In recent years, information regarding cancer prevention and treatment has improved. However, some of the information that is spread can be misleading or inaccurate. Becoming more aware of correct known risk factors as well as incorrect cancer myths may allow individuals to make more informed decisions regarding their health.

Cancer prevention- myths and facts

The list below expands on some of the most-researched known risk factors for cancer. It will also address common misconceptions and questions regarding each risk factor.

Smoking

People who use tobacco products or are regularly around environmental smoke (otherwise known as second-hand smoke) have an increased risk of developing cancer. This is because tobacco products have many chemicals inside them that directly damage DNA.

Is there use in quitting if a person has been smoking for a long time?
Yes. The damage done by smoking is cumulative, meaning that the longer an individual smokes, the higher their risk of developing cancer becomes. However, quitting at any age brings immediate benefits. Ten years after quitting, former smokers cut their risk of developing lung cancer by up to 50%.

Alcohol

Multiple research studies have found an association between heavy alcohol consumption and cancer. The more alcohol a person drinks regularly over time, the higher their risk of developing cancer will be.

Can drinking red wine help prevent cancer?
Most likely, no. Research studies have found that certain properties of red wine, such as resveratrol, may have anticancer properties. However, subsequent trials on humans have not shown any convincing evidence that these properties are effective in preventing or treating cancer.

Radiation

Radiation of particular wavelengths, called ionizing radiation, has enough energy to directly damage DNA and lead to cancer. This includes forms of high-energy radiation such as radon, ex-rays, and gamma rays.

Do cell phones and microwaves cause cancer?
No. Lower-energy, non-ionizing forms of radiation, such as the light and energy given off from cell phones, power lines, and microwaves does not damage DNA and has not been found to cause cancer.

Sunlight

The sun, tanning booths, and sun beds all give off ultraviolet (UV) radiation. Exposure to these UV rays can cause skin damage and increase the risk of getting skin cancer.

Are people with naturally olive or dark skin at risk of getting skin cancer?

Yes. Although people with lighter skin have an increased risk of getting skin cancer, anyone can burn if the sun is strong enough. The darker an individual's skin, the more melanin pigment they have, which protects the skin from UV rays, reducing their cancer risk. However, this does not mean that people with darker skin cannot get skin cancer. Often, people with darker skin will develop skin cancer on parts of their body that aren't usually exposed to the sun, such as on their palms or on the soles of their

Diet

Maintaining a healthy body weight and eating a well-balanced and healthy diet can reduce the risk of many cancers. Research has shown that regular consumption of fruits, vegetables, and high fibre foods can prevent the onset of cancer. In addition, frequent consumption of red and processed meats and salt can increase the risk of developing cancer.

Do artificial sweeteners cause cancer?

No. There are many stories in the media about various foods that are claimed to increase or decrease the risk of cancer. Some of these associations, however, do not have enough scientific evidence to support them.

For example, there is no evidence that the artificial sweeteners that are currently in the market are related to cancer risk in humans. Questions relating to artificial sweeteners and cancer risk came out in the 1980s when a study found a link between cyclamate (a type of artificial sweetener) and bladder cancer in laboratory rats. However, since that time, a large body of research strongly suggests that artificial sweeteners do not cause cancer in
