# Supplementary material for: Development of a tool to assess beliefs about mythical causes of cancer: the Cancer Awareness Measure Mythical Causes Scale
Source: BMJ Open. 2018 Dec 14;8(12):e022825. doi: 10.1136/bmjopen-2018-022825 (PMC6303629; doi:10.1136/bmjopen-2018-022825)
Supplement: Author's manuscript [file bmjopen-2018-022825.draft_revisions.pdf]

BMJ Open is committed to open peer review. As part of this commitment we make the peer review history of every article we publish publicly available.

When an article is published we post the peer reviewers' comments and the authors' responses online. We also post the versions of the paper that were used during peer review. These are the versions that the peer review comments apply to.

The versions of the paper that follow are the versions that were submitted during the peer review process. They are not the versions of record or the final published versions. They should not be cited or distributed as the published version of this manuscript.

BMJ Open is an open access journal and the full, final, typeset and author-corrected version of record of the manuscript is available on our site with no access controls, subscription charges or pay-per-view fees (http://bmjopen.bmj.com).

If you have any questions on BMJ Open's open peer review process please email info.bmjopen@bmj.com

BMJ Open

The development of a tool to assess beliefs about mythical causes of cancer: the Cancer Awareness Measure Mythical Causes Scale

Journal:	*BMJ Open*
Manuscript ID	bmjopen-2018-022825
Article Type:	Research
Date Submitted by the Author:	09-Mar-2018

Complete List of Authors:	Smith, Sam; University of Leeds, Leeds Institute of Health Sciences<br>Beard, Emma; UCL,<br>McGowan, Jennifer; University College London Research Department of Epidemiology and Public Health<br>Fox, Emma; Northwestern university, Division of General Internal Medicine<br>Cook, Chloe; Freelance<br>Pal, Radhika; University College London Research Department of Epidemiology and Public Health<br>Waller, Jo; UCL, Epidemiology and Public Health<br>Shahab, Lion; UCL, Dept of Epidemiology and Public Health

Keywords:	cancer, cancer beliefs, measurement, cancer myths

SCHOLARONE™
Manuscripts

For peer review only

The development of a tool to assess beliefs about mythical causes of cancer: the Cancer Awareness Measure Mythical Causes Scale

Samuel G. Smith[1,2], Emma Beard[2,3], Jennifer A. McGowan[2], Emma Fox[4], Chloe Cook[5], Radhika Pal[2], Jo Waller[2], Lion Shahab[2]

[1] Leeds Institute of Health Sciences, University of Leeds, Leeds, UK, LS2 9NL

[2] Research Department of Behavioural Science and Health, University College London, London, UK, WC1E 6BT

[3] Research Department of Clinical, Educational and Health Psychology, University College London, London, UK, WC1E 6BT

[4] Division of General Internal Medicine, Northwestern University, Chicago IL 60611

[5] Shift Design, St Johns Street, London, EC1M 4NJ

Corresponding author:

Dr Samuel Smith, Leeds Institute of Health Sciences, University of Leeds, Floor 10, Worsley Building, Leeds, LS2 9JT, Tel: 0113 343 0892, E: s.smith1@leeds.ac.uk

Abstract: 237/250

Manuscript: 3026/4000 (excluding abstract, figures, tables, legends and references)

Figures and tables: 4 /5

References: 23

Funding: This work was supported by a Cancer Research UK / Bupa Foundation Innovation Award (C42785/A20811). Smith and Waller are supported by Cancer Research UK Fellowships (C42785/A17965 and C7492/A17219 respectively).

For peer review only

ABSTRACT

Objectives: We aimed to develop a reliable and valid measure to assess public beliefs in mythical causes of cancer: the Cancer Awareness Measure - MYthical Causes Scale (CAM-MYCS).

Design and setting: Cancer myth items were generated from a literature review, social media and interviews (n=16). The CAM-MYCS was prepared by reducing items using a) an online sample (n=527) with exploratory factor analysis and b) cancer experts with DELPHI methodology (n=13). To assess test-retest reliability and sensitivity to change, students (n=91) completed the CAM-MYCS at baseline and 1-week after exposure to information on lifestyle-related cancer causes or control information. Construct validity was tested by comparing CAM-MYCS scores between cancer experts (n=25) and students (n=91). Factor structure and internal reliability were investigated in a national sample (n=1990).

Results: Out of 42 items generated, 12 were retained based on factor loadings, prevalence of endorsement, and expert consensus. CAM-MYCS scores improved among students exposed to information on cancer causes compared with the control group ($p<0.001$) and showed high test–retest reliability ($r=0.90$, $p<0.001$). Cancer experts reported higher CAM-MYCS scores than students ($p<0.001$). The factor structure of the CAM-MYCS was confirmed in the national sample and internal reliability was high ($\alpha=0.86$). Inclusion of the CAM-MYCS alongside items assessing knowledge of actual cancer causes did not affect responses.

Conclusions: The CAM-MYCS tool is a reliable and valid tool assessing beliefs in mythical causes of cancer, and it can be used alongside the existing CAM measure.

Keywords: cancer; cancer beliefs; measurement; cancer myths

For peer review only - http://bmjopen.bmj.com/site/about/guidelines.xhtml

Strengths and limitations of this study

- This is the first study to develop a valid and reliable tool for assessing public beliefs in mythical causes of cancer- The Cancer Awareness Measure - MYthical Causes Scale (CAM-MYCS).
- Mythical beliefs is a novel construct that could influence cancer prevention initiatives and the CAM-MYCS can be used to evaluate the success of cancer awareness campaigns
- While the CAM-MYCS was developed using iterative mixed-methods, it is possible that it does not reflect all common beliefs in mythical causes of cancer held by the public.
- Future studies are needed to develop cancer site-specific versions and explore variation in mythical beliefs between countries.

For peer review only

INTRODUCTION

A number of environmental causes of cancer have been identified including smoking, alcohol consumption, overweight, physical inactivity and poor diet.[1,2] An estimated 40% of cancer cases could be avoided through optimal adherence to lifestyle and environmental factors.[3] General population studies suggest awareness of environmental and lifestyle causes of cancer is mixed, which may undermine efforts to change behaviour at a population level.[4-8]

Recognition of prominent causes of cancer such as smoking and use of sunbeds is generally high.[4,5,9,10] However, other lifestyle factors such as alcohol consumption, overweight and low fruit and vegetable intake are poorly recognised. Awareness of the role of lifestyle factors in cancer is particularly poor among men, lower socioeconomic status groups, ethnic minorities and people with lower levels of education.[5,7-9,11,12] A survey of Dutch urinary bladder cancer patients shows the likelihood of attributing a cancer diagnosis to lifestyle factors is low, even among patients with known risk factors such as smoking.[13]

In addition to poor recognition of established causes of cancer, a sizeable minority of the public continue to endorse mythical causes for which there is no scientific evidence e.g. powerlines, deodorant and stress.[8,11,14,15] The majority of this work has been done in the United States and the UK. At present, there is no reliable and validated tool to assess beliefs in mythical causes of cancer. Current research employs unsystematic approaches when deciding which myths to include as distractor items in surveys, or uses open-ended assessments that measure recall rather than recognition.[11,14] The Cancer Awareness Measure (CAM)[16] and the Awareness and Beliefs about Cancer (ABC) measure[17] do not include items assessing awareness of mythical causes of cancer.

We aimed to a) identify beliefs about mythical causes of cancer held within the general public and b) develop a reliable and valid tool to measure belief in these mythical causes: the Cancer Awareness Measure Mythical Causes Scale (CAM-MYCS). The purpose of the measure is to identify and report the prevalence of belief in mythical risks; a factor that may be associated with health behaviour

For peer review only

choices. This new measure will help characterise the population and has the potential to lead to tailored interventions aimed at debunking mythical beliefs.

For peer review only

MATERIALS AND METHODS

Item generation

Systematic review

In May 2015 we searched for quantitative and qualitative articles reporting beliefs in mythical causes of cancer in general population samples (Appendix 1). Searches were run in Medline, EMBASE, PsycINFO, and PsycEXTRA. English text, peer-reviewed studies were included if they reported at least one myth about cancer causes from a general population sample largely (>50%) unaffected by cancer. We sought reports of non-cancer populations to ensure the items generated were relevant to the general population, which is the intended group for the CAM-MYCS tool. Studies were excluded if they only reported knowledge about true causes of cancer and did not measure cancer myths, were not peer-reviewed (e.g. commentaries, editorials), or were not written in the English language. A researcher (RP) retrieved all beliefs about mythical causes of cancer from the articles, and included them in an item pool.

Semi-structured interviews

A market research agency recruited 16 participants from the UK general population for semi-structured interviews. People were approached using purposive sampling, balanced across key socio-demographic variables (age, gender, ethnicity, and occupation). Participants who consented to take part in the study were invited to an interview at University College London or Queen Mary University of London. A topic guide was designed to explore beliefs about mythical cancer causes among the general public (Appendix 2). Each interview lasted approximately 30 minutes. Participants received £40 each for their time. Interviews were tape recorded and transcribed verbatim. A researcher trained in qualitative methods (RP) reviewed the interview data for beliefs about mythical cancer causes, and added them to the item pool.

Social media

Online newspaper articles with 'cancer' in the title or text, or that were indexed as 'cancer' reported between March and June 2015 were extracted using a Lexis Nexis search. The search was restricted to

For peer review only - http://bmjopen.bmj.com/site/about/guidelines.xhtml

four news agencies representing the full political spectrum and both broadsheet and tabloid newspapers (Independent, The Times, The Daily Mail, The Mirror). The BBC online news website was also included because it is widely accessed and is apolitical. The online comments associated with each article were extracted, and were the focus of the data analysis.

Between July and August 2015 we also extracted tweets related to perceived causes of cancer from the social media site 'Twitter' (http://www.twitter.com). We used the search terms 'cancer' AND ['cause' OR 'prevent' OR 'treat'] in an open-access software tool.[18] Tweets had to be produced by users with no commercial affiliation. Passive replicated messages (i.e. re-tweets) were excluded. A researcher (CC) recorded the frequency of beliefs about mythical cancer causes within the online newspaper comments and tweets using content analysis.[19] Due to the volume of beliefs reported, we only included those which were reported at least ten times.

Patient and public involvement

To identify further factors from a patient perspective, we recruited four individuals on to a patient and public involvement (PPI) panel. The representatives were a mixture of cancer survivors and relatives of cancer survivors recruited from a charity. Four individuals attended a presentation about our work, and provided suggestions which were accommodated within the item pool.

Item refinement

Researcher revision

Three researchers (SS, LS, JM) assessed the items to ensure there was no known association with cancer by using the latest evidence[1] and literature searches for each item. Similar items were combined (e.g. physical trauma/sports trauma). Items were removed using the following criteria: 1) an inability to be tested for a relationship with cancer (e.g. fate), 2) evidence of a causal route to cancer (e.g. hygiene), and 3) the suggested relationship for the item was cancer prevention rather than cause (e.g. eating avocados). This created a pool of 42 approved items.

For peer review only - http://bmjopen.bmj.com/site/about/guidelines.xhtml

DELPHI analysis

A DELPHI analysis was undertaken with experts (n=13). DELPHI analysis involves the anonymous collection of data from experts originating from a range of backgrounds, aimed at developing an unbiased consensus.[20] The panel included experts in oncology, public health, primary care, and behavioural science; all with professional interests in cancer.

In the first round experts were asked to individually list all beliefs about mythical cancer causes they were aware of. In round two, the list from round one was combined with the previously generated item pool. The experts identified items they believed were commonly endorsed myths about cancer causes in the general population. They were then asked to provide a list of the top ten items which they felt were most frequently endorsed by the public. In round 3, the experts viewed a list of the most commonly approved items from round 2 and indicated items they felt should be excluded. The experts also viewed a list of items that were previously suggested for removal and were asked to identify any they felt should be kept in the final questionnaire. This resulted in a final consensus of items.

Online survey

The aim of the online survey was to produce data for an exploratory factor analysis. The survey was done in parallel with the DELPHI analysis using the pool of 42 items. An online panel (N=500) was recruited through a research agency. Non-respondents and those with incomplete responses were removed (n=27), resulting in 473 useable respondents. Participants were asked to complete a questionnaire consisting of demographic information and the 42 items identified in the item generation stage. Eleven correct cancer causes from the CAM were also included.[16]

Respondents were asked, ‘How much do you agree that each of these can increase a person’s chance of developing cancer?’ Responses to each item were dichotomised into ‘correct’ (‘disagree’ or ‘strongly disagree’) and ‘incorrect’ (‘agree’, ‘strongly agree’ or ‘not sure’) responses. The order of the

For peer review only - http://bmjopen.bmj.com/site/about/guidelines.xhtml

items was randomised for each participant, such that incorrect and correct items were not shown separately.

Items underwent principal component analysis using varimax rotation in SPSS v24. To reduce the number of items, we observed the item loadings as well as the frequency with which myths were endorsed. Our a priori criteria for excluding items were: 1) failure to load strongly onto a single factor (loadings <.4) to ensure internal validity; 2) items for which more than 85% or fewer than 15% of the participants gave either a correct or incorrect response, to ensure sufficient variance in data and avoid ceiling effects;[21] and 3) items recommended for exclusion by the DELPHI analysis to ensure construct validity.

The final scoring of the CAM-MYCS was designed such that higher scores reflect superior knowledge about mythical cancer causes, i.e. one point was allocated for each myth that was correctly identified (i.e. ‘strongly disagree’ and ‘disagree’ responses). These scores were transformed to a score of 0-100 using the percent of maximum possible method.[22]

Item validation

Sensitivity to change and known groups

We recruited 91 students from University College London studying non-medical subjects using an e-newsletter. Twenty-one responses were excluded because of missing data, leaving data from 70 respondents. Participation was incentivised with entry into a prize draw for a £25 voucher. In an online survey, participants answered the 12-item CAM-MYCS measure at baseline and at 1-week follow-up. After completing the baseline questionnaire, the sample was randomised 1:1 to either the intervention or control group. The intervention group was sent an online link to a brief educational intervention describing general information regarding cancer development, the link between cancer and lifestyle behaviours, and commonly-held myths about cancer causes (Appendix 3). The control group did not receive any intervention.

To assess sensitivity to change, total CAM-MYCS scores for the intervention and control groups were compared at follow-up using repeated-measures ANOVA. Test-retest reliability of the CAM-MYCS measure was assessed by calculating Pearson's correlation coefficient for baseline and 1-week follow-up CAM-MYCS scores in the control condition.

Cancer experts (oncology nurses, scientists, cancer charity workers) were recruited through professional networks (n=25). The experts were invited via email, which included a link to a survey containing basic background information and the CAM-MYCS items. To determine construct validity, total CAM-MYCS scores for the students and experts were compared using repeated measures ANOVA and independent t-tests.[23] Analyses were done in SPSS version 24.0.

National survey

Data were from the Attitudes and Beliefs about Cancer-UK Survey (ABACUS), a nationally representative population-based cross-sectional survey in England (N=1,990). The survey was done between January and March 2016. This survey creates sample points using the 2001 Census small-area statistics and the Postcode Address File (stratified by social grade and Government Office Region) for random location sampling. Quotas for age, gender, children in the home, and working status were set for each location. Data were collected using computer-assisted face-to-face interviews in the respondents' homes.

Participants completed demographic information, the CAM risk factor measure[16] and the finalised CAM-MYCS measure. The CAM measure contains 11 known cancer causes and uses the same responses categories as the CAM-MYCS measure. Respondents were randomised to complete the CAM alone or the CAM and the CAM-MYCS measure on a 1:2 basis. A randomised design was used to investigate whether the CAM-MYCS measure could be used alongside the CAM, without affecting responses. Similar CAM scores in each group would indicate that the inclusion of the CAM-MYCS measure did not affect responses. Overall, 1,348 respondents were randomised to the CAM-MYCS and CAM condition, and 642 respondents to CAM questions alone. Participants were excluded if they

did not respond to all CAM-MYCS or CAM questions or if they used the same response for all items. This resulted in a sample size of 1,327 for CAM-MYCS and CAM and 640 for CAM alone.

Refusal rates were calculated to assess acceptability of the measure. Other missing data were handled using case-wise (or 'full information') maximum likelihood estimation. Using data from people who completed both the CAM and CAM-MYCS measures, we undertook a confirmatory factor analysis. This was done using the "Lavaan" package in R version 3.3.1.[24] The following fit statistics were computed: the Bayesian Information Criterion (BIC) and the Root Mean Square Error of Approximation (RMSEA). A two-factor model with CAM and CAM-MYCS items loading appropriately onto the respective factors would indicate construct validity. Statistical significance was set at $p<0.05$ in all studies.

Ethical approval

University ethical approval was gained for all studies. All participants consented to participate.

view only

RESULTS

Item generation

A total of 999 studies were identified in the systematic review. After the removal of duplicates, article screening and quality assessment, 16 studies were included. Fifty nine beliefs about mythical cancer causes were identified (Figure 1). The qualitative interview study yielded 33 beliefs. A total of 33,557 tweets and online comments were reviewed, of which 671 met inclusion criteria. From these, 93 beliefs about mythical cancer causes were identified. The PPI panel identified four beliefs about mythical cancer causes. After the review, interviews, PPI panel, and social media analysis, 103 unique beliefs about mythical cancer causes were included in the item pool.

Item refinement

Following our inclusion and exclusion criteria, we reduced the item pool to 42 beliefs about mythical causes of cancer (Table 1). The DELPHI analysis yielded no additional items. After three survey rounds, the expert group reached consensus on 13 items, which were recommended for inclusion in the measure.

Data from an online survey including the 42 item pool complemented the DELPHI analysis. The study sample are described in Table 2. In an exploratory principal component analysis, a one-component model was observed using both eigenfactor and scree-plot based criteria. All except three items (exposure to parabens, using illegal drugs, exposure to chemtrails) loaded strongly onto this factor (loadings $\geq$.40) and therefore further item selection was based on a priori criteria. No items were removed because of insufficient or excessive correct responses. Twenty-five items were removed because too few respondents endorsed the belief (i.e. answered incorrectly). Of the remaining items, two were removed (eating food containing sugar, using energy efficient lightbulbs) as these were not endorsed by expert consensus in the DELPHI analysis. This resulted in a final list of twelve items to be included in the CAM-MYCS tool (Table 1).

Item validation

For peer review only - http://bmjopen.bmj.com/site/about/guidelines.xhtml

Sensitivity to change and known groups

There were no differences in baseline scores for non-medical students (Intervention group: M=46.5, SD=26.8 vs. control group: M=48.0, SD=25.5; t(68)=0.24, p=0.81). However, only non-medical students who received information on lifestyle-related cancer causes after baseline assessment improved their CAM-MYCS scores at follow-up compared with non-medical students in the control condition (F(1,68)=18.47; p<.001), indicating the measure is sensitive to change (Intervention group: M=62.0, SD=31.0 vs. control group: M=41.4, SD=27.6; t(68)=2.94, p=0.005). Test-retest reliability was high for the control group completing the CAM-MYCS measure at baseline and 1-week follow-up (r=0.90, p<0.001).

The average CAM-MYCS scores for cancer experts was higher than the non-medical students at baseline (M=78.3, SD=24.4; vs. M=47.3, SD=26.0; t(93)=5.22, p<.001). This indicates the CAM-MYCS successfully distinguishes between groups known to have different levels of knowledge.

National survey

The group completing both the CAM-MYCS and CAM measures were similar to those completing the CAM measure only (Table 2). The CAM-MYCS measure had a low refusal rate (3.9%), indicating acceptable length and content. Responses were normally distributed, with a skewness of 0.25 (SE=0.07) and a kurtosis of 0.19 (SE=0.13), suggesting it captures a range of knowledge within the population. Mean CAM scores were comparable between the groups, demonstrating the inclusion of the CAM-MYCS items did not affect responses to the CAM (CAM + CAM-MYCS: M=52.78, SD=24.60 vs. CAM only: M=52.32, SD=23.79, p=0.65).

Confirmatory factor analysis including both CAM and CAM-MYCS items suggested a two-factor solution provided a better fit than a one-factor model (difference $\chi^2(1)=1302.6$, p<0.001). Items belonging to the CAM and CAM-MYCS measures loaded onto the appropriate factors (Table 3). The CAM-MYCS model was significantly improved following the removal of the item 'physical trauma'

For peer review only

(difference $\chi^2(1)$=71.52, p<0.001), yielding good fit statistics (BIC=38709.9, GFI=0.992, RMSEA=0.060). However, we decided to keep the item in the final measure because it was frequently identified within the item development phase, and good model fit was still observed when it was included (BIC=42450.6, GFI=0.992, RMSEA=0.054). The final 12-item CAM-MYCS measure had good internal reliability (Cronbach's α=0.86).

For peer review only

DISCUSSION

In this iterative set of studies we successfully identified commonly held beliefs about mythical causes of cancer and developed a valid and reliable measure to assess this construct. A range of perspectives were included to generate the items, including beliefs expressed on social media, in individual interviews, and by a diverse set of experts. In a series of studies, twelve items emerged as providing optimal fit in both exploratory and confirmatory factor analyses. The inclusion of the CAM-MYCS items alongside the CAM assessment did not influence awareness of actual cancer causes. Both item sets loaded appropriately onto the hypothesised factors in confirmatory factor analysis. We therefore encourage the two assessments to be used alongside each other to provide a more accurate assessment of public knowledge about cancer risk.

Evaluations of public awareness campaigns rely on assessments that only include established lifestyle behaviours related to cancer development.[25] The CAM-MYCS can be embedded within these evaluations, and these data can inform the content of the information disseminated within such campaigns. Population surveys investigating the prevalence of beliefs about mythical causes of cancer use assessments that are not validated. Progress can now be made in reliably and accurately assessing public beliefs in mythical causes of cancer.

Separately, we have reported the prevalence of CAM-MYCS items, their sociodemographic correlates and tested for associations with cancer prevention behaviours.[26] Understanding the extent to which mythical beliefs improve or undermine attempts to change health behaviours could inform the development of cancer prevention interventions and public health strategies. Finally, this measure has implications for assessing the beliefs of people diagnosed with cancer. Failure to attribute cancer to known risk factors is a recognised phenomenon,[13] and the extent to which people associate mythical factors with their own diagnosis is not known. Such studies can now be reliably undertaken, and have the potential to inform patient and provider dialogue.

For peer review only

These studies had limitations. While a range of different approaches were used to develop the item pool, each source may contain a biased sample. It is therefore possible that the CAM-MYCS measure does not reflect all common beliefs in mythical causes of cancer held by the public. Beliefs about risk factors may vary by cancer site;[6,10,13] this may not be captured by the CAM-MYCS scale and site-specific versions could be developed in the future. The online sample had a higher prevalence of young, female, White British and highly educated individuals than would be expected. This may affect generalisability of these findings. Mythical beliefs may emerge over time, and therefore a revision of the CAM-MYCS may be needed in the future. Lastly, this work was done in UK samples. The prevalence of beliefs in mythical cancer causes may vary across countries. Such beliefs are likely to be influenced strongly by culture and social environments, and international variation should be tested in future analyses.[27,28]

Conclusions

We used a series of iterative studies to demonstrate the reliability and validity of the CAM-MYCS measure in assessing beliefs in mythical causes of cancer among the general public. Of importance to public awareness campaigns and the evaluation of interventions, the CAM-MYCS measure can be used alongside the CAM without concerns that responses will be affected. This approach is recommended as it is likely to provide a more accurate assessment of public knowledge about cancer aetiology than current strategies.

For peer review only

ACKNOWLEDGEMENTS

We would like to thank the ABACUS study team for their support in conducting the survey.

AUTHOR CONTRIBUTION STATEMENT

SS and LS conceived the original idea for this study. SS and LS obtained funding. JM, SGS and LS managed the day-to-day running of the study. LS undertook the data analyses and wrote the initial draft with further input from all authors. LS is guarantor for this article. LS, JM, JW and SGS, reviewed and approved the final version. All researchers listed as authors are independent from the funders and all final decisions about the research were taken without constraint by the investigators. LS had full access to all the data in the study and had final responsibility for the decision to submit for publication.

FUNDING

This work was supported by a Cancer Research UK / Bupa Foundation Innovation Award (C42785/A20811). SS and JW are supported by Cancer Research UK Fellowships (C42785/A17965 and C7492/A17219 respectively). LS is a member of the UK Centre for Tobacco and Alcohol Studies (UKCTAS), funded under the auspices of the above UK Clinical Research Collaboration (MR/K023195/1). The funders had no role in the collection, analysis or interpretation of data; in the writing of the report; or in the decision to submit the article for publication

CONFLICT OF INTEREST

LS has received honoraria for talks, an unrestricted research grant and travel expenses to attend meetings and workshops by pharmaceutical companies that make smoking cessation products (Pfizer, Johnson&Johnson), and has acted as paid reviewer for grant awarding bodies and as a paid consultant for health care companies. Other research has been funded by the government, a community-interested company (National Centre for Smoking Cessation) and charitable sources.

PATIENT CONSENT

For peer review only

All participants consented to participate.

ETHICS APPROVAL

University College London ethical approval was gained for all studies.

DATA SHARING STATEMENT

Anonymised data will be shared upon request after all planned manuscripts have been published by the authors. A data sharing agreement will be generated and all data will be anonymised prior to sharing.

APPENDICES

Appendix 1. Systematic review terms

Appendix 2. Topic Guide

Appendix 3. Educational intervention

For peer review only

Figure Legends

Figure 1. Flow chart of CAM-MYCS development

For peer review only

Table 1. Items included in exploratory factor analysis (N=473)

Item	In final measure?	Recommended in Delphi?	Correct (%)	Unsure (%)	Incorrect (%)	Factor loading
Exposure to electromagnetic frequencies	Yes	Yes	22.8	43.6	33.6	0.400
Eating food containing additives	Yes	Yes	32.1	37.5	30.4	0.551
Living near power lines	Yes	Yes	32.8	38.4	28.8	0.481
Feeling stressed	Yes	Yes	41.6	31.1	27.3	0.541
Eating food containing artificial sweeteners	Yes	Yes	38.9	35.9	25.2	0.577
Using cleaning products	Yes	Yes	40.6	34.9	24.5	0.588
Eating genetically modified food	Yes	Yes	40.8	34.9	24.3	0.523
Using mobile phones	Yes	Yes	39.1	36.8	24.1	0.571
Using aerosol containers	Yes	Yes	44.8	31.7	23.5	0.640
Physical trauma, for example a punch or squeeze	Yes	Yes	49.9	28.3	21.8	0.561
Using microwave ovens	Yes	Yes	52.6	28.8	18.6	0.617
Drinking from plastic bottles	Yes	Yes	56.2	26.7	17.1	0.650
Exposure to parabens	No	Yes	15.6	52.7	31.7	0.291
Using illegal drugs	No	No	22.6	34.9	42.5	0.379
Exposure to chemtrails	No	No	20.9	44.2	34.9	0.380
Eating food containing sugar	No	No	59.2	23.7	17.1	0.671
Using energy efficient light bulbs	No	No	74.0	9.7	16.3	0.724
Using cosmetics	No	No	52.9	33.4	13.7	0.497
Breast development as a teenager	No	No	48.2	38.1	13.7	0.622
Experiencing anger	No	No	62.2	24.9	12.9	0.633
Exposing cancer to the air	No	No	61.5	25.6	12.9	0.546
Experiencing depression	No	No	57.9	29.4	12.7	0.611
Using or being exposed to incense	No	No	54.5	32.8	12.7	0.619
Undergoing surgery	No	No	59.4	28.3	12.3	0.591
Exposure to wifi signal	No	No	57.5	30.9	11.6	0.660
Using sunscreen	No	No	69.6	19.0	11.4	0.557
Eating dairy products	No	No	68.5	20.9	10.6	0.675
Using tampons	No	No	65.1	24.8	10.1	0.596
Using blood pressure medications	No	No	57.5	34.3	8.2	0.668
Using talcum powder	No	No	54.1	37.7	8.2	0.610
Frequent sexual activity with the same partner	No	No	79.1	13.1	7.8	0.596
Wearing tight clothing	No	No	69.8	22.4	7.8	0.638
Eating carbohydrates	No	No	71.5	20.9	7.6	0.709
Wearing an underwired bra	No	No	71.0	21.4	7.6	0.710

For peer review only

Carrying money in your bra	No	No	77.4	15.4	7.2	0.674
Receiving vaccinations	No	No	69.8	23.0	7.2	0.714
Consuming vitamin pills	No	No	68.9	24.3	6.8	0.646
Eating chewing gum	No	No	73.6	20.1	6.3	0.648
Experiencing jet lag	No	No	74.8	19.1	6.1	0.727
Eating food containing gluten	No	No	69.3	24.6	6.1	0.745
Exposure to the cold	No	No	77.4	16.9	5.7	0.685
Eating food containing soya	No	No	71.0	23.3	5.7	0.722

Italics indicate parameter that do not meet pre-defined inclusion criteria

For peer review only

Table 2. Participant characteristics for studies

	Online Panel Study	Student and experts validation survey		National survey		
	N=498	Cancer Experts (n=25)	Non-medical students (n=70)	Overall (N=1967)	CAM-MYCS and CAM (N=1327)	CAM alone (N=640)
Age						
Mean (SD)	46.1 (88.4)	38.2 (10.8)	24.7 (7.3)	43.7 (16.0)	43.9 (15.9)	43.4 (16.1)
≤30	31.5 (156)	32.0 (8)	84.3 (59)	27.6 (566)	27.4 (363)	28.7 (184)
31-40	17.3 (86)	36.0 (9)	11.4 (8)	18.5 (378)	17.6 (233)	18.9 (121)
41-50	18.5 (92)	12.0 (3)	1.4 (1)	16.2 (332)	17.5 (232)	13.9 (89)
51-60	18.1 (90)	16.0 (4)	2.9 (2)	17.5 (359)	17.0 (225)	19.4 (124)
61+	14.5 (72)	4.0 (1)	0 (0)	20.2 (412)	20.6 (274)	19.1 (122)
Gender						
Male	39.2 (195)	8.0 (2)	25.7 (18)	46.8 (921)	46.0 (610)	48.6 (311)
Female	60.6 (302)	92.0 (23)	74.3 (52)	53.2 (1046)	54.0 (717)	51.4 (329)
Prefer not to say	0.2 (1)	0 (0)	0 (0)	0 (0)	0 (0)	0 (0)
Ethnicity						
White British	82.1 (409)	76.0 (19)	28.6 (20)	75.6 (1548)	76.1 (1010)	75.9 (486)
White Other	5.4 (27)	12.0 (3)	28.6 (20)	8.4 (173)	8.2 (109)	8.8 (56)
Other	12.5 (62)	12.0 (3)	42.8 (30)	16.0 (327)	15.7 (2.08)	15.3 (98)
Education						
Degree or higher	36.1 (180)	80.0 (20)	77.1 (54)	26.0 (533)	28.1 (373)	23.0 (147)
Higher education	9.0 (45)	12.0 (3)	8.6 (6)	11.2 (229)	11.6 (154)	11.1 (71)
A-Level^	21.1 (105)	4.0 (1)	14.3 (10)	13.7 (281)	14.1 (187)	13.8 (88)
ONC/BTEC	4.0 (20)	0 (0)	0 (0)	6.1 (125)	5.9 (78)	7.0 (45)
GCSE/O-Level	23.7 (118)	0 (0)	0 (0)	25.0 (513)	24.5 (325)	25.8 (165)
None	4.0 (20)	0 (0)	0 (0)	13.8 (282)	12.4 (164)	15.0 (96)
Other	0.8 (4)	4.0 (1)	0 (0)	2.8 (58)	2.5 (33)	3.8 (24)
Prefer not to say	1.2 (6)	0 (0)	0 (0)	1.3 (27)	1.0 (13)	0.6 (4)

Note: Figures reported are % (n) for all apart from mean age (SD). ^ For the student and expert validation survey, this category also included secondary education diplomas.

For peer review only - http://bmjopen.bmj.com/site/about/guidelines.xhtml

Table 3. Unstandardized and standardised loadings for confirmatory models (N=1327)

	Single Factor model			Two factor model					
	Factor 1			Factor 1 (CAM)			Factor 2 (CAM-MYCS)		
	Unstandardized		Standardised	Unstandardized		Standardised	Unstandardized		Standardised
	Estimate	SE	Estimate	Estimate	SE	Estimate	Estimate	SE	Estimate
CAM 1	1	0	0.506	1	0	0.543			
CAM 2	1.098	0.055	0.584	1.067	0.05	0.608			
CAM 3	1.2	0.057	0.651	1.204	0.052	0.7			
CAM 4	1.156	0.056	0.625	1.096	0.051	0.636			
CAM 5	1.063	0.055	0.553	1.077	0.051	0.6			
CAM 6	1.046	0.049	0.662	1.076	0.046	0.73			
CAM 7	1.165	0.057	0.615	1.177	0.052	0.667			
CAM 8	1.247	0.059	0.665	1.148	0.052	0.656			
CAM 9	1.179	0.057	0.638	1.109	0.051	0.644			
CAM 10	1.142	0.054	0.667	1.162	0.05	0.727			
CAM 11	1.297	0.06	0.677	1.274	0.055	0.712			
CAM-MYCS 1	1.177	0.06	0.669				1	0	0.69
CAM-MYCS 2	1.356	0.065	0.742				1.12	0.044	0.746
CAM-MYCS 3	1.207	0.062	0.665				1.056	0.043	0.705
CAM-MYCS 4	1.253	0.066	0.644				1.004	0.047	0.626
CAM-MYCS 5	1.278	0.063	0.703				1.074	0.044	0.717
CAM-MYCS 6	1.127	0.059	0.647				0.937	0.042	0.652
CAM-MYCS 7	1.165	0.061	0.653				1.043	0.043	0.708
CAM-MYCS 8	1.259	0.064	0.676				1.09	0.045	0.71
CAM-MYCS 9	1.208	0.06	0.693				1.022	0.042	0.713
CAM-MYCS 10	0.967	0.059	0.526				0.811	0.044	0.532
CAM-MYCS 11	1.168	0.061	0.65				1.046	0.043	0.706
CAM-MYCS 12	1.13	0.059	0.653				0.971	0.042	0.68

Note: For the two factor model the covariance between the two factors was 0.352 ($p<0.001$), CAM – Cancer Awareness Measure; CAM-MYCS – Cancer Awareness Measure Mythical Causes Scale

For peer review only

REFERENCES

[1] International Agency for Research on Cancer. IARC Monographs on the evaluation of carcinogenic risks to humans. 2017.

[2] World Cancer Research Fund International / American Institute for Cancer Research. Continuous Update Project: Diet, nutrition, physical activity and the prevention of cancer. Summary of strong evidence. 2017.

[3] Lagerlund M, Hvidberg L, Hajdarevic S, Fischer Pedersen A, Runesdotter S, Vedsted P, et al. Awareness of risk factors for cancer: a comparative study of Sweden and Denmark. BMC Public Health 2015;15:1156. doi:10.1186/s12889-015-2512-9.

[4] Hvidberg L, Pedersen AF, Wulff CN, Vedsted P. Cancer awareness and socio-economic position: results from a population-based study in Denmark. BMC Cancer 2014;14:581. doi:10.1186/1471-2407-14-581.

[5] Simon AE, Juszczyk D, Smyth N, Power E, Hiom S, Peake MD, et al. Knowledge of lung cancer symptoms and risk factors in the UK: development of a measure and results from a population-based survey. Thorax 2012;67:426–432.

[6] Sanderson SC, Waller J, Jarvis MJ, Humphries SE, Wardle J. Awareness of lifestyle risk factors for cancer and heart disease among adults in the UK. Patient Educ Couns 2009;74:221–7. doi:10.1016/j.pec.2008.08.003.

[7] Redeker C, Wardle J, Wilder D, Hiom S, Miles A. The launch of Cancer Research UK's "Reduce the Risk" campaign: baseline measurements of public awareness of cancer risk factors in 2004. Eur J Cancer Oxf Engl 1990 2009;45:827–36. doi:10.1016/j.ejca.2008.10.018.

[8] Cancer Research UK. Cancer Awareness Measure (CAM). Key Findings Report; 2014 & Trends Analysis (2008-2014). London, UK: 2016.

[9] Marlow L a. V, Robb KA, Simon AE, Waller J, Wardle J. Awareness of cancer risk factors among ethnic minority groups in England. Public Health 2012;126:702–9. doi:10.1016/j.puhe.2012.05.005.

[10] Wardle J, Waller J, Brunswick N, Jarvis MJ. Awareness of risk factors for cancer among British adults. Public Health 2001;115:173–4. doi:10.1038/sj/ph/1900752.

[11] Hawkins NA, Berkowitz Z, Peipins LA. What does the public know about preventing cancer? Results from the Health Information National Trends Survey (HINTS). Health Educ Behav Off Publ Soc Public Health Educ 2010;37:490–503. doi:10.1177/1090198106296770.

[12] Stein K, Zhao L, Crammer C, Gansler T. Prevalence and sociodemographic correlates of beliefs regarding cancer risks. Cancer 2007;110:1139–48. doi:10.1002/cncr.22880.

[13] Stubbings S, Robb KA, Waller J, Ramirez A, Austoker J, Macleod U, et al. Development of a measurement tool to assess public awareness of cancer. Br J Cancer 2009;101:S13–S17.

[14] Simon AE, Forbes LJL, Boniface D, Warburton F, Brain KE, Dessaix A, et al. An international measure of awareness and beliefs about cancer: development and testing of the ABC. BMJ Open 2012;2:e001758. doi:10.1136/bmjopen-2012-001758.

[15] McGrath R. Twython. 2013. Accessed online: https://twython.readthedocs.io/en/latest/# 11th October, 2017.

[16] Hsieh H-F, Shannon SE. Three Approaches to Qualitative Content Analysis. Qual Health Res 2005;15:1277–88. doi:10.1177/1049732305276687.

[17] Pill J. The Delphi method: Substance, context, a critique and an annotated bibliography. Socioecon Plann Sci 1971;5:57–71. doi:10.1016/0038-0121(71)90041-3.

[18] Kline P. Handbook of Psychological Testing, Second Edition. 2 edition. London ; New York: Routledge; 1999.

[19] Cohen P, Cohen J, Aiken LS, West SG. The Problem of Units and the Circumstance for POMP. Multivar Behav Res 1999;34:315–46. doi:10.1207/S15327906MBR3403_2.

[20] DeVellis RF. Scale Development: Theory and Applications. Third edition. Thousand Oaks, Calif: SAGE Publications, Inc; 2011.

[21] Rosseel Y. lavaan: An R Package for Structural Equation Modeling. J Stat Softw 2012.

[22] Smith SG, Osborne K, Tring S, George H, Power E. Evaluating the impact of a community-based cancer awareness roadshow on awareness, attitudes and behaviors. Prev Med 2016;87:138–43. doi:10.1016/j.ypmed.2016.02.034.

For peer review only

[23] Shahab L, McGowan J, Waller J, Smith SG. Prevalence of beliefs about actual and mythical causes of cancer and their association with socio-demographic and health-related characteristics: findings from a national cross-sectional survey. Submitted.
[24] Morse EP, Maegga B, Joseph G, Miesfeldt S. Breast Cancer Knowledge, Beliefs, and Screening Practices among Women Seeking Care at District Hospitals in Dar es Salaam, Tanzania. Breast Cancer Basic Clin Res 2014;8:73–9. doi:10.4137/BCBCR.S13745.
[25] Olanyinka A, Joel A, Feyisayo G. Knowledge and beliefs about breast cancer among female undergraduate in a selected private tertiary institution in Edo State Nigeria. Int J Trop Med 2012;7:151–6.

For peer review only

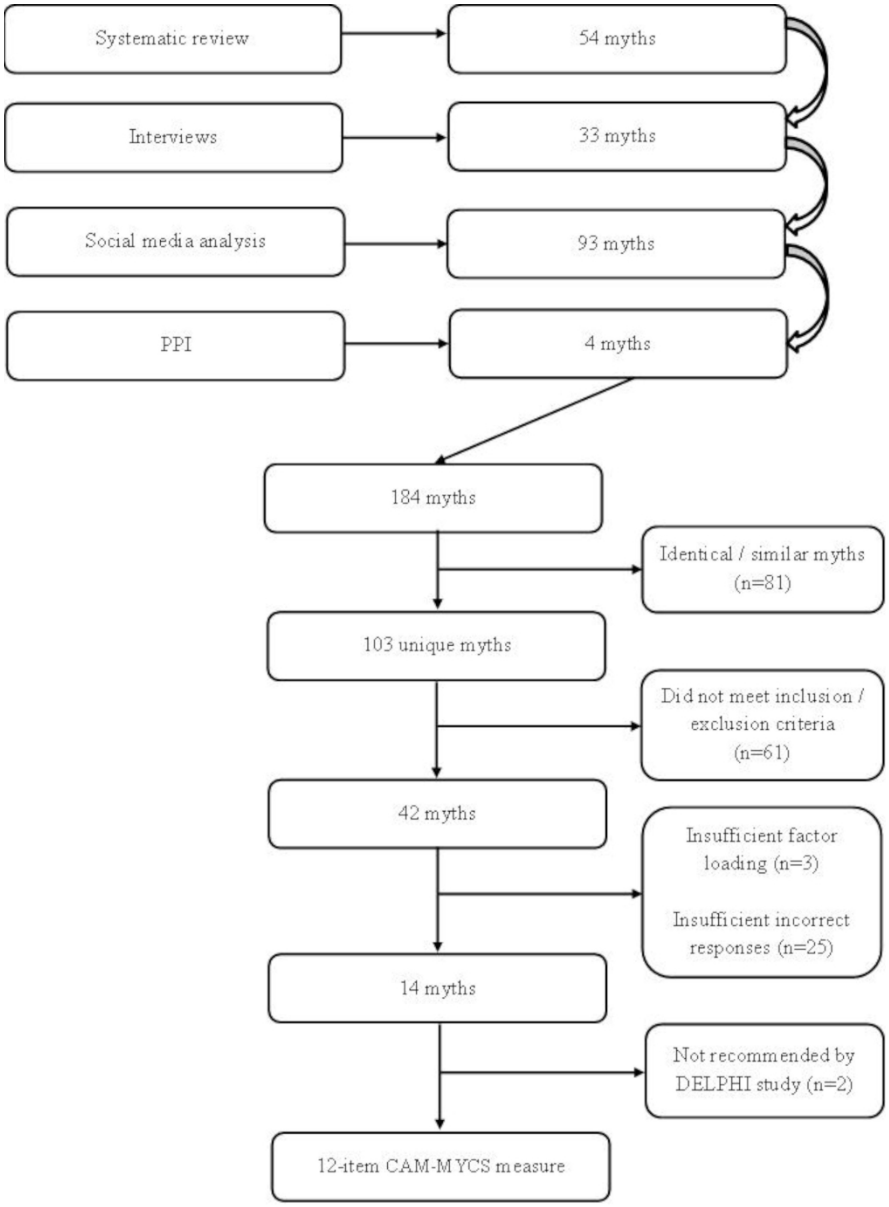

Flow chart of CAM-MYCS development

177x241mm (300 x 300 DPI)

Supplementary Table 1. Citation generated from search terms, May 2015

Step	Term Category	Search string	Medline/ EMBASE	PsycINFO/ EXTRA	All together-deduplicated
1	"Cancer" terms^	(cancer OR neoplasm).me	2,776,856	47,990	
2	"Belief" terms^	(caus* or attribute* or belie* or perce* or (illness adj representation*) or know* or aware* or thought* or idea*).me	697,570	1,386,598	
3	"Wrong" terms	(false* or incorrect* or myth* or erroneous* or wrong* or mistaken* or error or untrue or fallacious).mp	921,613	125,798	
4	"Cancer patient" terms	cancer adj patient*.tw	302,437	11,606	
5	Combination	1 AND 2 AND 3	784	534	
6	Exclusion	5 NOT 4	716	460	
7	Limits	Human, English	632	417	**999**

^Mesh terms/Subject heading terms for Medline/Embase only (otherwise .tw)

For peer review only

Supplementary information

Appendix A: Topic guide

SEMI-STRUCTURED INTERVIEW SCHEDULE (CANCER AWARENESS)

Objective
In-depth exploration of the incorrect causal beliefs about cancer in the UK general population sample and identifying existing socioeconomic inequalities.

To generate items to develop a valid and reliable tool for assessing cancer awareness in the public.

Beginning the interview
- Introduction of researcher; topics to be covered/structure of interview; confidentiality; timing; confirming consent for tape-recording

1.1 Background
- Name RP, a masters' student in Health Psychology at UCL
- Interested in understanding and knowing about people's understanding of cancer. Don't worry there are no right or wrong answers. We are just interested in your ideas.

1.2 Interview Outline
- If that's ok, I will ask you a couple of questions about your views on cancer in general,
- Also interested to hear about your beliefs about the different causes of cancer
- At the end will describe a new cancer awareness assessment tool we are developing at UCL and I would very much appreciate to hear your views about that tool

1.3 Interview Arrangements
- Should take around half an hour, but we might find lots to talk about, so please go into as much detail as possible.
- Ok to record interview (consent)
- Of course it's all completely anonymous/confidential and recordings will be transcribed

During the interview (main themes to be covered)

2.1. Questions exploring the causal beliefs or risk perceptions of cancer:

2.1.1. How much do you understand about cancer? Have you have had any experience with cancer through friends or relatives?

2.1.2. A number of different things have been linked to the development of cancer, but new discoveries are often being made. What do you think are the most common causes of cancer?

For peer review only

2.1.3. What things do you think affects a person's chances of developing cancer?

2.1.4. What are the best ways to avoid getting cancer?

2.1.5. Do you think that certain behaviours or risk factors are more important for specific people (e.g. prompt: men vs. women, younger vs. older; different ethnic groups)?

2.1.6 We have been asking all our interviewees this question, what do you think our chances of developing cancer is in your lifetime? Would you say it is higher or lower compared to an average person?

Prompts for questions:

- "What else?"
- "Take your time to think about it"

3. Ending the interview

- Anything else participants would like to say?
- Any important issues not raised?
- Any other questions regarding research?
- Debriefing about study
- Provision of researcher (RP) and principal researcher's (LS/SS) contact information, should anything else come up
- Thanks

For peer review only

LONDON'S GLOBAL UNIVERSITY

UCL

Cancer Information Leaflet

Known risk factors and common myths

IBAC Study Educational Leaflet

By: Emma Fox

What is cancer?

Cancer is the name of a collection of diseases where the cells of the body begin to divide abnormally. These cells can become invasive, and grow out of control, spreading into surrounding tissues. Many cancers grow to form solid masses of tissue, known as tumours.

Cancer can start almost anywhere in the human body, and there are more than 100 different types of cancer.

What causes cancer?

Changes to genes, called mutations, play a vital role in the development of cancer. Some of the gene changes that lead to cancer may be inherited. Most gene mutations, however, happen after birth.

It is not usually possible to say exactly how a person develops cancer. Nevertheless, research has shown that certain risk factors may increase the likelihood of an individual developing cancer. These include factors such as exposure to ultraviolet rays found in sunlight, as well as lifestyle behaviours such as smoking and heavy alcohol consumption. Other risk factors include family history and age.

Myths and misconceptions about cancer

In recent years, information regarding cancer prevention and treatment has improved. However, some of the information that is spread can be misleading or inaccurate. Becoming more aware of correct known risk factors as well as incorrect cancer myths may allow individuals to make more informed decisions regarding their health.

For peer review only

Cancer prevention- myths and facts

The list below expands on some of the most-researched known risk factors for cancer. It will also address common misconceptions and questions regarding each risk factor.

Smoking

People who use tobacco products or are regularly around environmental smoke (otherwise known as second-hand smoke) have an increased risk of developing cancer. This is because tobacco products have many chemicals inside them that directly damage DNA.

Is there use in quitting if a person has been smoking for a long time?

Yes. The damage done by smoking is cumulative, meaning that the longer an individual smokes, the higher their risk of developing cancer becomes. However, quitting at any age brings immediate benefits. Ten years after quitting, former smokers cut their risk of developing lung cancer by up to 50%.

Alcohol

Multiple research studies have found an association between heavy alcohol consumption and cancer. The more alcohol a person drinks regularly over time, the higher their risk of developing cancer will be.

Can drinking red wine help prevent cancer?

Most likely, no. Research studies have found that certain properties of red wine, such as resveratrol, may have anticancer properties. However, subsequent trials on humans have not shown any convincing evidence that these properties are effective in preventing or treating cancer.

Radiation

Radiation of particular wavelengths, called ionizing radiation, has enough energy to directly damage DNA and lead to cancer. This includes forms of high-energy radiation such as radon, ex-rays, and gamma rays.

Do cell phones and microwaves cause cancer?

No. Lower-energy, non-ionizing forms of radiation, such as the light and energy given off from cell phones, power lines, and microwaves does not damage DNA and has not been found to cause cancer.

For peer review only - http://bmjopen.bmj.com/site/about/guidelines.xhtml

Sunlight

The sun, tanning booths, and sun beds all give off ultraviolet (UV) radiation. Exposure to these UV rays can cause skin damage and increase the risk of getting skin cancer.

Are people with naturally olive or dark skin at risk of getting skin cancer?

Yes. Although people with lighter skin have an increased risk of getting skin cancer, anyone can burn if the sun is strong enough. The darker an individual's skin, the more melanin pigment they have, which protects the skin from UV rays, reducing their cancer risk. However, this does not mean that people with darker skin cannot get skin cancer. Often, people with darker skin will develop skin cancer on parts of their body that aren't usually exposed to the sun, such as on their palms or on the soles of their

Diet

Maintaining a healthy body weight and eating a well-balanced and healthy diet can reduce the risk of many cancers. Research has shown that regular consumption of fruits, vegetables, and high fibre foods can prevent the onset of cancer. In addition, frequent consumption of red and processed meats and salt can increase the risk of developing cancer.

Do artificial sweeteners cause cancer?

No. There are many stories in the media about various foods that are claimed to increase or decrease the risk of cancer. Some of these associations, however, do not have enough scientific evidence to support them.

For example, there is no evidence that the artificial sweeteners that are currently in the market are related to cancer risk in humans. Questions relating to artificial sweeteners and cancer risk came out in the 1980s when a study found a link between cyclamate (a type of artificial sweetener) and bladder cancer in laboratory rats. However, since that time, a large body of research strongly suggests that artificial sweeteners do not cause cancer in

For peer review only

STROBE 2007 (v4) Statement—Checklist of items that should be included in reports of *cross-sectional studies*

Section/Topic	Item #	Recommendation	Reported on page #
**Title and abstract**	1	(*a*) Indicate the study's design with a commonly used term in the title or the abstract	1
		(*b*) Provide in the abstract an informative and balanced summary of what was done and what was found	2
**Introduction**			
Background/rationale	2	Explain the scientific background and rationale for the investigation being reported	4-5
Objectives	3	State specific objectives, including any prespecified hypotheses	4-5
**Methods**			
Study design	4	Present key elements of study design early in the paper	6-10
Setting	5	Describe the setting, locations, and relevant dates, including periods of recruitment, exposure, follow-up, and data collection	6-10
Participants	6	(*a*) Give the eligibility criteria, and the sources and methods of selection of participants	6-10
Variables	7	Clearly define all outcomes, exposures, predictors, potential confounders, and effect modifiers. Give diagnostic criteria, if applicable	6-10
Data sources/ measurement	8*	For each variable of interest, give sources of data and details of methods of assessment (measurement). Describe comparability of assessment methods if there is more than one group	6-10
Bias	9	Describe any efforts to address potential sources of bias	*10-11*
Study size	10	Explain how the study size was arrived at	6-10
Quantitative variables	11	Explain how quantitative variables were handled in the analyses. If applicable, describe which groupings were chosen and why	9-11
Statistical methods	12	(*a*) Describe all statistical methods, including those used to control for confounding	9-11
		(*b*) Describe any methods used to examine subgroups and interactions	9-11
		(*c*) Explain how missing data were addressed	9-11
		(*d*) If applicable, describe analytical methods taking account of sampling strategy	9-11
		(*e*) Describe any sensitivity analyses	9-11
**Results**			

Participants	13*	(a) Report numbers of individuals at each stage of study—eg numbers potentially eligible, examined for eligibility, confirmed eligible, included in the study, completing follow-up, and analysed	12-14
		(b) Give reasons for non-participation at each stage	12-14
		(c) Consider use of a flow diagram	Figure
Descriptive data	14*	(a) Give characteristics of study participants (eg demographic, clinical, social) and information on exposures and potential confounders	12 (tables)
		(b) Indicate number of participants with missing data for each variable of interest	12 (tables)
Outcome data	15*	Report numbers of outcome events or summary measures	12-14 (tables)
Main results	16	(*a*) Give unadjusted estimates and, if applicable, confounder-adjusted estimates and their precision (eg, 95% confidence interval). Make clear which confounders were adjusted for and why they were included	12-14
		(*b*) Report category boundaries when continuous variables were categorized	6-10
		(*c*) If relevant, consider translating estimates of relative risk into absolute risk for a meaningful time period	N/A
Other analyses	17	Report other analyses done—eg analyses of subgroups and interactions, and sensitivity analyses	N/A
**Discussion**			
Key results	18	Summarise key results with reference to study objectives	15-16
Limitations	19	Discuss limitations of the study, taking into account sources of potential bias or imprecision. Discuss both direction and magnitude of any potential bias	15-16
Interpretation	20	Give a cautious overall interpretation of results considering objectives, limitations, multiplicity of analyses, results from similar studies, and other relevant evidence	15-16
Generalisability	21	Discuss the generalisability (external validity) of the study results	15-16
**Other information**			
Funding	22	Give the source of funding and the role of the funders for the present study and, if applicable, for the original study on which the present article is based	17

*Give information separately for cases and controls in case-control studies and, if applicable, for exposed and unexposed groups in cohort and cross-sectional studies.

Note: An Explanation and Elaboration article discusses each checklist item and gives methodological background and published examples of transparent reporting. The STROBE checklist is best used in conjunction with this article (freely available on the Web sites of PLoS Medicine at http://www.plosmedicine.org/, Annals of Internal Medicine at http://www.annals.org/, and Epidemiology at http://www.epidem.com/). Information on the STROBE Initiative is available at www.strobe-statement.org.

For peer review only

BMJ Open

The development of a tool to assess beliefs about mythical causes of cancer: the Cancer Awareness Measure Mythical Causes Scale

Journal:	*BMJ Open*
Manuscript ID	bmjopen-2018-022825.R1
Article Type:	Research
Date Submitted by the Author:	09-Aug-2018
Complete List of Authors:	Smith, Sam; University of Leeds, Leeds Institute of Health Sciences<br>Beard, Emma; UCL,<br>McGowan, Jennifer; University College London Research Department of Epidemiology and Public Health<br>Fox, Emma; Northwestern university, Division of General Internal Medicine<br>Cook, Chloe; Freelance<br>Pal, Radhika; University College London Research Department of Epidemiology and Public Health<br>Waller, Jo; UCL, Epidemiology and Public Health<br>Shahab, Lion; UCL, Dept of Epidemiology and Public Health
**Primary Subject Heading**:	Oncology
Secondary Subject Heading:	Oncology, Public health, Qualitative research, Epidemiology, Communication
Keywords:	cancer, cancer beliefs, measurement, cancer myths

SCHOLARONE™
Manuscripts

The development of a tool to assess beliefs about mythical causes of cancer: the Cancer Awareness Measure Mythical Causes Scale

Samuel G. Smith[1,2], Emma Beard[2,3], Jennifer A. McGowan[2], Emma Fox[4], Chloe Cook[5], Radhika Pal[2], Jo Waller[2], Lion Shahab[2]

[1] Leeds Institute of Health Sciences, University of Leeds, Leeds, UK, LS2 9NL

[2] Research Department of Behavioural Science and Health, University College London, London, UK, WC1E 6BT

[3] Research Department of Clinical, Educational and Health Psychology, University College London, London, UK, WC1E 6BT

[4] Division of General Internal Medicine, Northwestern University, Chicago IL 60611

[5] Shift Design, St Johns Street, London, EC1M 4NJ

Corresponding author:

Dr Samuel Smith, Leeds Institute of Health Sciences, University of Leeds, Floor 10, Worsley Building, Leeds, LS2 9JT, Tel: 0113 343 0892, E: s.smith1@leeds.ac.uk

Abstract: 237/250

Manuscript: 3026/4000 (excluding abstract, figures, tables, legends and references)

Figures and tables: 4 /5

References: 23

Funding: This work was supported by a Cancer Research UK / Bupa Foundation Innovation Award (C42785/A20811). Smith and Waller are supported by Cancer Research UK Fellowships (C42785/A17965 and C7492/A17219 respectively).

For peer review only

ABSTRACT

Objectives: We aimed to develop a reliable and valid measure to assess public beliefs in mythical causes of cancer: the Cancer Awareness Measure - MYthical Causes Scale (CAM-MYCS).

Design and setting: Cancer myth items were generated from a literature review, social media and interviews (n=16). The CAM-MYCS was prepared by reducing items using a) an online sample (n=527) with exploratory factor analysis and b) cancer experts with DELPHI methodology (n=13). To assess test-retest reliability and sensitivity to change, students (n=91) completed the CAM-MYCS at baseline and 1-week after exposure to information on lifestyle-related cancer causes or control information. Construct validity was tested by comparing CAM-MYCS scores between cancer experts (n=25) and students (n=91). Factor structure and internal reliability were investigated in a national sample (n=1993).

Results: Out of 42 items generated, 12 were retained based on factor loadings, prevalence of endorsement, and expert consensus. CAM-MYCS scores improved (fewer myths endorsed) among students exposed to information on cancer causes compared with the control group ($p<0.001$) and showed high test–retest reliability ($r=0.90$, $p<0.001$). Cancer experts reported higher CAM-MYCS scores (fewer myths endorsed) than students ($p<0.001$). The factor structure of the CAM-MYCS was confirmed in the national sample and internal reliability was high ($\alpha=0.86$). Inclusion of the CAM-MYCS alongside items assessing knowledge of actual cancer causes did not affect responses.

Conclusions: The CAM-MYCS tool is a reliable and valid tool assessing beliefs in mythical causes of cancer, and it can be used alongside items assessing known causes of cancer.

Keywords: cancer; cancer beliefs; measurement; cancer myths

For peer review only

Strengths and limitations of this study

- This is the first study to develop a valid and reliable tool for assessing public beliefs in mythical causes of cancer- The Cancer Awareness Measure - MYthical Causes Scale (CAM-MYCS).
- Mythical beliefs is a novel construct that could influence cancer prevention initiatives and the CAM-MYCS can be used to evaluate the success of cancer awareness campaigns
- While the CAM-MYCS was developed using iterative mixed-methods, it is possible that it does not reflect all common beliefs in mythical causes of cancer held by the public.
- Future studies are needed to develop cancer site-specific versions and explore variation in mythical beliefs between countries.

For peer review only

INTRODUCTION

A number of environmental causes of cancer have been identified including smoking, alcohol consumption, overweight, physical inactivity and poor diet.[1,2] An estimated 40% of cancer cases could be avoided through optimal adherence to lifestyle and environmental factors.[3] General population studies suggest awareness of environmental and lifestyle causes of cancer is mixed, which may undermine efforts to change behaviour at a population level.[4–9]

Recognition of prominent causes of cancer such as smoking and use of sunbeds is generally high.[5,6,10,11] However, other lifestyle factors such as alcohol consumption, overweight and low fruit and vegetable intake are poorly recognised. Awareness of the role of lifestyle factors in cancer is particularly poor among men, lower socioeconomic status groups, ethnic minorities and people with lower levels of education.[6,8–10,12,13] A survey of Dutch urinary bladder cancer patients shows the likelihood of attributing a cancer diagnosis to lifestyle factors is low, even among patients with known risk factors such as smoking.[14]

In addition to poor recognition of established causes of cancer, a sizeable minority of the public continue to endorse mythical causes for which there is no scientific consensus for a causal effect e.g. powerlines, deodorant and stress.[9–11] The majority of this work has been done in the United States and the UK. It is important to investigate the public's causal beliefs about cancer as the way in which we think about disease risk factors can affect treatment decision-making and prevention behaviour.[15–17] Understanding how common such mythical beliefs are among the general population can help to guide campaigns attempting to improve public understanding of cancer.

At present, there is no reliable and validated tool to assess beliefs in mythical causes of cancer. Current research employs unsystematic approaches when deciding which myths to include as distractor items in surveys, or uses open-ended assessments that measure recall rather than recognition.[10,11] The Cancer Awareness Measure (CAM)[18] and the Awareness and Beliefs about Cancer (ABC) measure [19] are the most frequently used validated assessments of known risk factor

For peer review only

awareness, however they do not include items assessing awareness of mythical causes of cancer. Therefore only awareness of known risk factors is assessed in population surveys and public health campaign evaluations [20]. Assessing belief in mythical causes of cancer may provide a complementary perspective in which to study the effects of public understanding of cancer on treatment decision-making and lifestyle behaviours.

We aimed to a) identify beliefs about mythical causes of cancer held within the general public and b) develop a reliable and valid tool to measure belief in these mythical causes: the Cancer Awareness Measure Mythical Causes Scale (CAM-MYCS). The purpose of the measure is to identify and report the prevalence of belief in mythical risks, i.e. currently unsubstantiated risks for cancer; a factor that may be associated with health behaviour choices. This new measure will help characterise the population and has the potential to lead to tailored interventions aimed at debunking mythical beliefs.

For peer review only

MATERIALS AND METHODS

Item generation

Systematic review

In May 2015 we searched for quantitative and qualitative articles reporting beliefs in mythical causes of cancer in general population samples (Appendix 1). Searches were run in Medline, EMBASE, PsycINFO, and PsycEXTRA. English text, peer-reviewed studies were included if they reported at least one myth about cancer causes from a general population sample largely (>50%) unaffected by cancer. We sought reports of non-cancer populations to ensure the items generated were relevant to the general population, which is the intended group for the CAM-MYCS tool. Studies were excluded if they only reported knowledge about true causes of cancer and did not measure cancer myths, were not peer-reviewed (e.g. commentaries, editorials), or were not written in the English language. A researcher (RP) retrieved all beliefs about mythical causes of cancer from the articles, and included them in an item pool.

Semi-structured interviews

A market research agency recruited 16 participants from the UK general population for semi-structured interviews. People were approached using purposive sampling, balanced across key socio-demographic variables (age, gender, ethnicity, and occupation). Participants who consented to take part in the study were invited to an interview at University College London or Queen Mary University of London. A topic guide was designed to explore beliefs about mythical cancer causes among the general public (Appendix 2). Each interview lasted approximately 30 minutes. Participants received £40 each for their time. Interviews were tape recorded and transcribed verbatim. A researcher trained in qualitative methods (RP) reviewed the interview data for beliefs about mythical cancer causes, and added them to the item pool.

Social media

Online newspaper articles with ‘cancer’ in the title or text, or that were indexed as ‘cancer’ reported between March and June 2015 were extracted using a Lexis Nexis search. The search was restricted to

For peer review only

four news agencies representing the full political spectrum and both broadsheet and tabloid newspapers (Independent, The Times, The Daily Mail, The Mirror). The BBC online news website was also included because it is widely accessed and is apolitical. The online comments associated with each article were extracted, and were the focus of the data analysis.

Between July and August 2015 we also extracted tweets related to perceived causes of cancer from the social media site 'Twitter' (http://www.twitter.com). We used the search terms 'cancer' AND ['cause' OR 'prevent' OR 'treat'] in an open-access software tool.[21] Tweets had to be produced by users with no commercial affiliation. Passive replicated messages (i.e. re-tweets) were excluded. A researcher (CC) recorded the frequency of beliefs about mythical cancer causes within the online newspaper comments and tweets using content analysis.[22] Due to the volume of beliefs reported, we only included those which were reported at least ten times.

Patient and public involvement

To identify further factors from a patient perspective, we recruited four individuals on to a patient and public involvement (PPI) panel. The representatives were a mixture of cancer survivors and relatives of cancer survivors recruited from a charity. Four individuals attended a presentation about our work, and provided suggestions which were accommodated within the item pool.

Item refinement

Researcher revision

In December 2015, three researchers (SS, LS, JM) used the latest scientific evidence[1] to examine the scientific consensus for the associations between each item and cancer. Similar items were combined (e.g. physical trauma/sports trauma). Items were removed using the following criteria: 1) an inability to be tested for a relationship with cancer (e.g. fate), 2) evidence of a causal route to cancer (e.g. hygiene), and 3) the suggested relationship for the item was cancer prevention rather than cause (e.g. eating avocados). This created a pool of 42 approved items.

For peer review only - http://bmjopen.bmj.com/site/about/guidelines.xhtml

DELPHI analysis

A DELPHI analysis was undertaken with experts (n=13). DELPHI analysis involves the anonymous collection of data from experts originating from a range of backgrounds, aimed at developing an unbiased consensus.[23] The panel included experts in oncology, public health, primary care, and behavioural science; all with professional interests in cancer.

In the first round experts were asked to individually list all beliefs about mythical cancer causes they were aware of. In round two, the list from round one was combined with the previously generated item pool. The experts identified items they believed were commonly endorsed myths about cancer causes in the general population. They were then asked to provide a list of the top ten items which they felt were most frequently endorsed by the public. In round 3, the experts viewed a list of the most commonly approved items from round 2 and indicated items they felt should be excluded. The experts also viewed a list of items that were previously suggested for removal and were asked to identify any they felt should be kept in the final questionnaire. This resulted in a final consensus of items.

Online survey

The aim of the online survey was to produce data for an exploratory factor analysis. The survey was done in parallel with the DELPHI analysis using the pool of 42 items. An online panel (N=500) was recruited through a research agency. Non-respondents and those with incomplete responses were removed (n=27), resulting in 473 useable respondents. Participants were asked to complete a questionnaire consisting of demographic information and the 42 items identified in the item generation stage. Eleven correct cancer causes from the CAM were also included.[18]

Respondents were asked, ‘How much do you agree that each of these can increase a person’s chance of developing cancer?’ Responses to each item were dichotomised into ‘correct’ (‘disagree’ or ‘strongly disagree’) and ‘incorrect’ (‘agree’, ‘strongly agree’ or ‘not sure’) responses. The order of the

items was randomised for each participant, such that incorrect and correct items were not shown separately.

Items underwent principal component analysis using varimax rotation in SPSS v24. To reduce the number of items, we observed the item loadings as well as the frequency with which myths were endorsed. Our a priori criteria for excluding items were: 1) failure to load strongly onto a single factor (loadings <.4) to ensure internal validity; 2) items for which more than 85% or fewer than 15% of the participants gave either a correct or incorrect response, to ensure sufficient variance in data and avoid ceiling effects;[24] and 3) items recommended for exclusion by the DELPHI analysis to ensure construct validity.

The final scoring of the CAM-MYCS was designed such that higher scores reflect superior knowledge about mythical cancer causes, i.e. one point was allocated for each myth that was correctly identified (i.e. 'strongly disagree' and 'disagree' responses). These scores were transformed to a score of 0-100 using the percent of maximum possible method.[25]

Item validation

Sensitivity to change and known groups

We recruited 91 students from University College London studying non-medical subjects using an e-newsletter. Twenty-one responses were excluded because of missing data, leaving data from 70 respondents. Participation was incentivised with entry into a prize draw for a £25 voucher. In an online survey, participants answered the 12-item CAM-MYCS measure at baseline and at 1-week follow-up. After completing the baseline questionnaire, the sample was randomised 1:1 to either the intervention or control group. The intervention group was sent an online link to a brief educational intervention describing general information regarding cancer development, the link between cancer and lifestyle behaviours, and commonly-held myths about cancer causes (Appendix 3). The control group did not receive any intervention.

To assess sensitivity to change, total CAM-MYCS scores for the intervention and control groups were compared at follow-up using repeated-measures ANOVA. Test-retest reliability of the CAM-MYCS measure was assessed by calculating Pearson's correlation coefficient for baseline and 1-week follow-up CAM-MYCS scores in the control condition.

Cancer experts (oncology nurses, scientists, cancer charity workers) were recruited through professional networks (n=25). The experts were invited via email, which included a link to a survey containing basic background information and the CAM-MYCS items. To determine construct validity, total CAM-MYCS scores for the students and experts were compared using repeated measures ANOVA and independent t-tests.[24] Analyses were done in SPSS version 24.0.

National survey

Data were from the Attitudes and Beliefs about Cancer-UK Survey (ABACUS), a nationally representative population-based cross-sectional survey in England (N=1,993). The survey was done between January and March 2016. This survey creates sample points using the 2001 Census small-area statistics and the Postcode Address File (stratified by social grade and Government Office Region) for random location sampling. Quotas for age, gender, children in the home, and working status were set for each location. Data were collected using computer-assisted face-to-face interviews in the respondents' homes.

Participants completed demographic information, the CAM risk factor measure [18] and the finalised CAM-MYCS measure. The CAM measure contains 11 known cancer causes and uses the same responses categories as the CAM-MYCS measure. Respondents were randomised to complete the CAM alone or the CAM and the CAM-MYCS measure on a 1:2 basis. A randomised design was used to investigate whether the CAM-MYCS measure could be used alongside the CAM, without affecting responses. Similar CAM scores in each group would indicate that the inclusion of the CAM-MYCS measure did not affect responses. Overall, 1,352 respondents were randomised to the CAM-MYCS and CAM condition, and 641 respondents to CAM questions alone. Participants were excluded if they

did not respond to all CAM-MYCS or CAM questions or if they used the same response for all items. This resulted in a sample size of 1,327 for CAM-MYCS and CAM and 640 for CAM alone. Those with missing data on CAM-MYCS or CAM were less likely to be of white ethnicity but there were no other differences.

Refusal rates were calculated to assess acceptability of the measure. Other missing data were handled using case-wise (or 'full information') maximum likelihood estimation. Using data from people who completed both the CAM and CAM-MYCS measures, we undertook a confirmatory factor analysis. This was done using the "Lavaan" package in R version 3.3.1.[26] The following fit statistics were computed: the Bayesian Information Criterion (BIC), the Goodness of Fit Index (GFI) and the Root Mean Square Error of Approximation (RMSEA). A two-factor model with CAM and CAM-MYCS items loading appropriately onto the respective factors would indicate construct validity. Statistical significance was set at $p<0.05$ in all studies.

Ethical approval

University ethical approval was gained for all studies. All participants consented to participate.

w only

RESULTS

Item generation

A total of 999 studies (k) were identified in the systematic review. Duplicates were removed (k=987 remaining), and titles and abstracts were screened (k=55 remaining). Following full text screening, 16 studies remained and underwent quality assessment using the Newcastle-Ottawa Scale [27]. Fifty nine beliefs about mythical cancer causes were identified (Figure 1). The qualitative interview study yielded 33 beliefs. A total of 33,557 tweets and online comments were reviewed, of which 671 met inclusion criteria. From these, 93 beliefs about mythical cancer causes were identified. The PPI panel identified four beliefs about mythical cancer causes. After the review, interviews, PPI panel, and social media analysis, 103 unique beliefs about mythical cancer causes were included in the item pool.

Item refinement

Following our inclusion and exclusion criteria, we reduced the item pool to 42 beliefs about mythical causes of cancer (Table 1). The DELPHI analysis yielded no additional items. After three survey rounds, the expert group reached consensus on 13 items, which were recommended for inclusion in the measure.

Data from an online survey including the 42 item pool complemented the DELPHI analysis. The study sample are described in Table 2. In an exploratory principal component analysis, a one-component model was observed using both eigenfactor and scree-plot based criteria. All except three items (exposure to parabens, using illegal drugs, exposure to chemtrails) loaded strongly onto this factor (loadings ≥.40) and therefore further item selection was based on a priori criteria. No items were removed because of insufficient or excessive correct responses. Twenty-five items were removed because too few respondents endorsed the belief (i.e. answered incorrectly). Of the remaining items, two were removed (eating food containing sugar, using energy efficient lightbulbs) as these were not endorsed by expert consensus in the DELPHI analysis. This resulted in a final list of twelve items to be included in the CAM-MYCS tool (Table 1).

Item validation

Sensitivity to change and known groups

There were no differences in baseline scores for non-medical students (Intervention group: M=46.5, SD=26.8 vs. control group: M=48.0, SD=25.5; t(68)=0.24, p=0.81). However, only non-medical students who received information on lifestyle-related cancer causes after baseline assessment improved their CAM-MYCS scores at follow-up compared with non-medical students in the control condition (F(1,68)=18.47; p<.001), indicating the measure is sensitive to change (Intervention group: M=62.0, SD=31.0 vs. control group: M=41.4, SD=27.6; t(68)=2.94, p=0.005). Test-retest reliability was high for the control group completing the CAM-MYCS measure at baseline and 1-week follow-up (r=0.90, p<0.001).

The average CAM-MYCS scores for cancer experts was higher than the non-medical students at baseline (M=78.3, SD=24.4; vs. M=47.3, SD=26.0; t(93)=5.22, p<.001). This indicates the CAM-MYCS successfully distinguishes between groups known to have different levels of knowledge.

National survey

The group completing both the CAM-MYCS and CAM measures were similar to those completing the CAM measure only (Table 2). The CAM-MYCS measure had a low refusal rate (3.9%), indicating acceptable length and content. Responses were normally distributed, with a skewness of 0.25 (SE=0.07) and a kurtosis of 0.19 (SE=0.13), suggesting it captures a range of knowledge within the population. Mean CAM scores were comparable between the groups, demonstrating the inclusion of the CAM-MYCS items did not affect responses to the CAM (CAM + CAM-MYCS: M=52.78, SD=24.60 vs. CAM only: M=52.32, SD=23.79, p=0.65).

Confirmatory factor analysis including both CAM and CAM-MYCS items suggested a two-factor solution provided a better fit than a one-factor model (difference $\chi^2(1)=1302.6$, p<0.001). Items belonging to the CAM and CAM-MYCS measures loaded onto the appropriate factors (Table 3). The

For peer review only - http://bmjopen.bmj.com/site/about/guidelines.xhtml

CAM-MYCS model was significantly improved following the removal of the item 'physical trauma' (difference $\chi^2(1)=71.52$, $p<0.001$), yielding good fit statistics (BIC=38709.9, GFI=0.992, RMSEA=0.067, 95% CI 0.065-0.070). However, we decided to keep the item in the final measure because it was frequently identified within the item development phase, and good model fit was still observed when it was included (BIC=42450.6, GFI=0.992, RMSEA=0.054, 95% CI 0.052 – 0.056). The final 12-item CAM-MYCS measure had good internal reliability (Cronbach's α=0.86).

For peer review only

DISCUSSION

In this iterative set of studies we successfully identified commonly held beliefs about mythical causes of cancer and developed a valid and reliable measure to assess this construct. A range of perspectives were included to generate the items, including beliefs expressed on social media, in individual interviews, and by a diverse set of experts. In a series of studies, twelve items emerged as providing optimal fit in both exploratory and confirmatory factor analyses. The inclusion of the CAM-MYCS items alongside the CAM assessment did not influence awareness of actual cancer causes. Both item sets loaded appropriately onto the hypothesised factors in confirmatory factor analysis. We therefore encourage the two assessments to be used alongside each other to provide a more accurate assessment of public knowledge about cancer risk.

Evaluations of public awareness campaigns rely on assessments that only include established lifestyle behaviours related to cancer development.[20] However, when such campaigns involve face-to-face interaction between healthcare professionals and the public, a portion of the conversations are likely to involve discussions about cancer myths. The CAM-MYCS can be embedded within these evaluations to examine if myths are being adequately addressed by such campaigns. Furthermore, these outcome data can inform the content of the written information disseminated within such campaigns. Until now, population surveys investigating the prevalence of beliefs about mythical causes of cancer have used assessments that are not validated. Progress can now be made in reliably and accurately assessing public beliefs in mythical causes of cancer.

Separately, we have reported the prevalence of CAM-MYCS items, their sociodemographic correlates and tested for associations with cancer prevention behaviours [28]. Briefly, participants showed poor awareness of factors not causally linked with cancer, with only a third of mythical cancer causes identified correctly. The most commonly endorsed cancer myths were stress (41.7%), food additives (41.1%), exposure to non-ionizing electromagnetic frequencies (34.7%), and genetically modified foods (34.1%). Perhaps counterintuitively, better awareness of mythical risk factors was associated

For peer review only

with a greater likelihood of smoking and having a higher aggregated behaviour risk score (composed of smoking, physical activity, overweight, fruit and vegetables and alcohol consumption).

Understanding the extent to which mythical beliefs improve or undermine attempts to change health behaviours could inform the development of cancer prevention interventions and public health strategies. This measure also has implications for assessing the beliefs of people diagnosed with cancer. Failure to attribute cancer to known risk factors is a recognised phenomenon,[18,29] and the extent to which people associate mythical factors with their own diagnosis is not known. Such studies can now be reliably undertaken, and have the potential to inform patient and provider dialogue.

There are limitations with the CAM-MYCS tool. Given the limitations of empiricism and the impossibility to prove a negative, we are unable to completely rule out causal relationships between the factors included within the tool and the development of cancer. The items were carefully chosen on the basis of scientific consensus, using reports from leading agencies [1], and experts from a range of relevant disciplines within the Delphi study. It is possible that future research investigating the effects of these factors on carcinogenesis will report findings to convince the scientific community of a likely causal relationship. For instance, there is some preliminary evidence of a weak association between certain forms of cancer and mobile phone use [30] and non-ionizing electromagnetic radiation more generally [31]. However, the extent to which this is causal is still debated [32]. If scientific consensus changes, the CAM-MYCS scale should be adapted to reflect the latest evidence. It is also important for users of the tool to note that we are referring to non-ionizing electromagnetic frequencies within the scale; and this should be distinguished from other forms of radiation known to cause cancer, such as ultraviolet radiation.

The studies involved in the development of the tool also have limitations. While a range of different approaches were used to develop the item pool, each source may contain a biased sample. It is therefore possible that the CAM-MYCS measure does not reflect all common beliefs in mythical causes of cancer held by the public. Beliefs about risk factors may vary by cancer site;[6,33,34] this

For peer review only

may not be captured by the CAM-MYCS scale and site-specific versions could be developed in the future. The online sample had a higher prevalence of young, female, White British and highly educated individuals than would be expected. This may affect generalisability of these findings. Mythical beliefs may emerge over time, and therefore a revision of the CAM-MYCS may be needed in the future. Lastly, this work was done in UK samples. The prevalence of beliefs in mythical cancer causes may vary across countries. Such beliefs are likely to be influenced strongly by culture and social environments, and international variation should be tested in future analyses.[35,36]

Conclusions

We used a series of iterative studies to demonstrate the reliability and validity of the CAM-MYCS measure in assessing beliefs in mythical causes of cancer among the general public. Of importance to public awareness campaigns and the evaluation of interventions, the CAM-MYCS measure can be used alongside the CAM without concerns that CAM responses will be affected. This approach is recommended as it is likely to provide a more accurate assessment of public knowledge about cancer aetiology than current strategies.

For peer review only

ACKNOWLEDGEMENTS

We would like to thank the ABACUS study team for their support in conducting the survey.

AUTHOR CONTRIBUTION STATEMENT

SS and LS conceived the original idea for this study. SS and LS obtained funding. JM, SS and LS managed the day-to-day running of the study. LS, SS, JM, CC, EF, and RP were involved in data collection (including the systematic review, interviews, social media analysis, and the student, online and national surveys). LS, EB and JM undertook the data analysis. SS wrote the initial draft with further input from EB, JM, EF, CC, RP, JW, and LS. LS is guarantor for this article. SS, EB, JM, EF, CC, RP, JW and LS reviewed and approved the final version. All researchers listed as authors are independent from the funders and all final decisions about the research were taken without constraint by the investigators. LS, EB and JM had full access to all the data in the study. SS and LS had final responsibility for the decision to submit for publication.

FUNDING

This work was supported by a Cancer Research UK / Bupa Foundation Innovation Award (C42785/A20811). SS and JW are supported by Cancer Research UK Fellowships (C42785/A17965 and C7492/A17219 respectively). LS is a member of the UK Centre for Tobacco and Alcohol Studies (UKCTAS), funded under the auspices of the above UK Clinical Research Collaboration (MR/K023195/1). The funders had no role in the collection, analysis or interpretation of data; in the writing of the report; or in the decision to submit the article for publication

CONFLICT OF INTEREST

LS has received honoraria for talks, an unrestricted research grant and travel expenses to attend meetings and workshops by pharmaceutical companies that make smoking cessation products (Pfizer, Johnson&Johnson), and has acted as paid reviewer for grant awarding bodies and as a paid consultant for health care companies. Other research has been funded by the government, a community-

For peer review only

interested company (National Centre for Smoking Cessation) and charitable sources. SS is an academic advisor for LUTO.

PATIENT CONSENT

All participants consented to participate.

ETHICS APPROVAL

University College London ethical approval was gained for all studies.

DATA SHARING STATEMENT

Anonymised data will be shared upon request after all planned manuscripts have been published by the authors. A data sharing agreement will be generated and all data will be anonymised prior to sharing.

APPENDICES

Appendix 1. Systematic review terms

Appendix 2. Topic Guide

Appendix 3. Educational intervention

For peer review only

Figure Legends

Figure 1. Flow chart of CAM-MYCS development

For peer review only

Table 1. Items included in exploratory factor analysis for CAM-MYCS (N=473)

Item	In final measure?	Recommended in Delphi?	Correct (%)	Unsure (%)	Incorrect (%)	Factor loading
Exposure to electromagnetic frequencies	Yes	Yes	22.8	43.6	33.6	0.400
Eating food containing additives	Yes	Yes	32.1	37.5	30.4	0.551
Living near power lines	Yes	Yes	32.8	38.4	28.8	0.481
Feeling stressed	Yes	Yes	41.6	31.1	27.3	0.541
Eating food containing artificial sweeteners	Yes	Yes	38.9	35.9	25.2	0.577
Using cleaning products	Yes	Yes	40.6	34.9	24.5	0.588
Eating genetically modified food	Yes	Yes	40.8	34.9	24.3	0.523
Using mobile phones	Yes	Yes	39.1	36.8	24.1	0.571
Using aerosol containers	Yes	Yes	44.8	31.7	23.5	0.640
Physical trauma, for example a punch or squeeze	Yes	Yes	49.9	28.3	21.8	0.561
Using microwave ovens	Yes	Yes	52.6	28.8	18.6	0.617
Drinking from plastic bottles	Yes	Yes	56.2	26.7	17.1	0.650
Exposure to parabens	No	Yes	15.6	52.7	31.7	0.291
Using illegal drugs	No	No	22.6	34.9	42.5	0.379
Exposure to chemtrails	No	No	20.9	44.2	34.9	0.380
Eating food containing sugar	No	No	59.2	23.7	17.1	0.671
Using energy efficient light bulbs	No	No	74.0	9.7	16.3	0.724
Using cosmetics	No	No	52.9	33.4	13.7	0.497
Breast development as a teenager	No	No	48.2	38.1	13.7	0.622
Experiencing anger	No	No	62.2	24.9	12.9	0.633
Exposing cancer to the air	No	No	61.5	25.6	12.9	0.546
Experiencing depression	No	No	57.9	29.4	12.7	0.611
Using or being exposed to incense	No	No	54.5	32.8	12.7	0.619
Undergoing surgery	No	No	59.4	28.3	12.3	0.591
Exposure to wifi signal	No	No	57.5	30.9	11.6	0.660
Using sunscreen	No	No	69.6	19.0	11.4	0.557
Eating dairy products	No	No	68.5	20.9	10.6	0.675
Using tampons	No	No	65.1	24.8	10.1	0.596
Using blood pressure medications	No	No	57.5	34.3	8.2	0.668
Using talcum powder	No	No	54.1	37.7	8.2	0.610
Frequent sexual activity with the same partner	No	No	79.1	13.1	7.8	0.596
Wearing tight clothing	No	No	69.8	22.4	7.8	0.638
Eating carbohydrates	No	No	71.5	20.9	7.6	0.709
Wearing an underwired bra	No	No	71.0	21.4	7.6	0.710
Carrying money in your bra	No	No	77.4	15.4	7.2	0.674

For peer review only

Receiving vaccinations	No	No	69.8	23.0	7.2	0.714
Consuming vitamin pills	No	No	68.9	24.3	6.8	0.646
Eating chewing gum	No	No	73.6	20.1	6.3	0.648
Experiencing jet lag	No	No	74.8	19.1	6.1	0.727
Eating food containing gluten	No	No	69.3	24.6	6.1	0.745
Exposure to the cold	No	No	77.4	16.9	5.7	0.685
Eating food containing soya	No	No	71.0	23.3	5.7	0.722

Electromagnetic frequencies refers to non-ionizing radiation of low and high frequencies such as WiFi and Radio/TV frequencies

For peer review only

Development of the Cancer Awareness Measure Mythical Causes Scale

Table 2. Participant characteristics for studies

	Online Panel Study	Student and experts validation survey		National survey		
	N=498	Cancer Experts (n=25)	Non-medical students (n=70)	Overall (N=1967)	CAM-MYCS and CAM (N=1327)	CAM alone (N=640)
Age						
Mean (SD)	42.2 (15.5)	38.2 (10.8)	24.7 (7.3)	43.7 (16.0)	43.9 (15.9)	43.4 (16.1)
≤30	31.5 (156)	32.0 (8)	84.3 (59)	27.6 (566)	27.4 (363)	28.7 (184)
31-40	17.3 (86)	36.0 (9)	11.4 (8)	18.5 (378)	17.6 (233)	18.9 (121)
41-50	18.5 (92)	12.0 (3)	1.4 (1)	16.2 (332)	17.5 (232)	13.9 (89)
51-60	18.1 (90)	16.0 (4)	2.9 (2)	17.5 (359)	17.0 (225)	19.4 (124)
61+	14.5 (72)	4.0 (1)	0 (0)	20.2 (412)	20.6 (274)	19.1 (122)
Gender						
Male	39.2 (195)	8.0 (2)	25.7 (18)	46.8 (921)	46.0 (610)	48.6 (311)
Female	60.6 (302)	92.0 (23)	74.3 (52)	53.2 (1046)	54.0 (717)	51.4 (329)
Prefer not to say	0.2 (1)	0 (0)	0 (0)	0 (0)	0 (0)	0 (0)
Ethnicity						
White British	82.1 (409)	76.0 (19)	28.6 (20)	75.6 (1548)	76.1 (1010)	75.9 (486)
White Other	5.4 (27)	12.0 (3)	28.6 (20)	8.4 (173)	8.2 (109)	8.8 (56)
Other	12.5 (62)	12.0 (3)	42.8 (30)	16.0 (327)	15.7 (2.08)	15.3 (98)
Education						
Degree or higher	36.1 (180)	80.0 (20)	77.1 (54)	26.0 (533)	28.1 (373)	23.0 (147)
Higher education	9.0 (45)	12.0 (3)	8.6 (6)	11.2 (229)	11.6 (154)	11.1 (71)
A-Level^	21.1 (105)	4.0 (1)	14.3 (10)	13.7 (281)	14.1 (187)	13.8 (88)
ONC/BTEC	4.0 (20)	0 (0)	0 (0)	6.1 (125)	5.9 (78)	7.0 (45)
GCSE/O-Level	23.7 (118)	0 (0)	0 (0)	25.0 (513)	24.5 (325)	25.8 (165)
None	4.0 (20)	0 (0)	0 (0)	13.8 (282)	12.4 (164)	15.0 (96)
Other	0.8 (4)	4.0 (1)	0 (0)	2.8 (58)	2.5 (33)	3.8 (24)
Prefer not to say	1.2 (6)	0 (0)	0 (0)	1.3 (27)	1.0 (13)	0.6 (4)

Note: Figures reported are % (n) for all apart from mean age (SD). ^ For the student and expert validation survey, this category also included secondary education diplomas.

Table 3. Unstandardized and standardised loadings for confirmatory models (N=1327)

	Single Factor model			Two factor model					
	Factor 1			Factor 1 (CAM)			Factor 2 (CAM-MYCS)		
	Unstandardized		Standardised	Unstandardized		Standardised	Unstandardized		Standardised
	Estimate	SE	Estimate	Estimate	SE	Estimate	Estimate	SE	Estimate
CAM 1	1	0	0.506	1	0	0.543			
CAM 2	1.098	0.055	0.584	1.067	0.05	0.608			
CAM 3	1.2	0.057	0.651	1.204	0.052	0.7			
CAM 4	1.156	0.056	0.625	1.096	0.051	0.636			
CAM 5	1.063	0.055	0.553	1.077	0.051	0.6			
CAM 6	1.046	0.049	0.662	1.076	0.046	0.73			
CAM 7	1.165	0.057	0.615	1.177	0.052	0.667			
CAM 8	1.247	0.059	0.665	1.148	0.052	0.656			
CAM 9	1.179	0.057	0.638	1.109	0.051	0.644			
CAM 10	1.142	0.054	0.667	1.162	0.05	0.727			
CAM 11	1.297	0.06	0.677	1.274	0.055	0.712			
CAM-MYCS 1	1.177	0.06	0.669				1	0	0.69
CAM-MYCS 2	1.356	0.065	0.742				1.12	0.044	0.746
CAM-MYCS 3	1.207	0.062	0.665				1.056	0.043	0.705
CAM-MYCS 4	1.253	0.066	0.644				1.004	0.047	0.626
CAM-MYCS 5	1.278	0.063	0.703				1.074	0.044	0.717
CAM-MYCS 6	1.127	0.059	0.647				0.937	0.042	0.652
CAM-MYCS 7	1.165	0.061	0.653				1.043	0.043	0.708
CAM-MYCS 8	1.259	0.064	0.676				1.09	0.045	0.71
CAM-MYCS 9	1.208	0.06	0.693				1.022	0.042	0.713
CAM-MYCS 10	0.967	0.059	0.526				0.811	0.044	0.532
CAM-MYCS 11	1.168	0.061	0.65				1.046	0.043	0.706
CAM-MYCS 12	1.13	0.059	0.653				0.971	0.042	0.68

Note: For the two factor model the covariance between the two factors was 0.352 ($p<0.001$), CAM – Cancer Awareness Measure; CAM-MYCS – Cancer Awareness Measure Mythical Causes Scale

For peer review only

REFERENCES

1 International Agency for Research on Cancer. IARC Monographs on the evaluation of carcinogenic risks to humans. 2017. Accessed online, http://monographs.iarc.fr/ENG/Classification/

2 World Cancer Research Fund International / American Institute for Cancer Research. Continuous Update Project: Diet, nutrition, physical activity and the prevention of cancer. Summary of strong evidence. 2017. www.wcrf.org/cupmatrix

3 Brown KF, Rumgay H, Dunlop C, *et al.* The fraction of cancer attributable to modifiable risk factors in England, Wales, Scotland, Northern Ireland, and the United Kingdom in 2015. *Br J Cancer* 2018;**118**:1130–41. doi:10.1038/s41416-018-0029-6

4 Lagerlund M, Hvidberg L, Hajdarevic S, *et al.* Awareness of risk factors for cancer: a comparative study of Sweden and Denmark. *BMC Public Health* 2015;**15**:1156. doi:10.1186/s12889-015-2512-9

5 Hvidberg L, Pedersen AF, Wulff CN, *et al.* Cancer awareness and socio-economic position: results from a population-based study in Denmark. *BMC Cancer* 2014;**14**:581. doi:10.1186/1471-2407-14-581

6 Simon AE, Juszczyk D, Smyth N, *et al.* Knowledge of lung cancer symptoms and risk factors in the UK: development of a measure and results from a population-based survey. *Thorax* 2012;**67**:426–432.

7 Sanderson SC, Waller J, Jarvis MJ, *et al.* Awareness of lifestyle risk factors for cancer and heart disease among adults in the UK. *Patient Educ Couns* 2009;**74**:221–7. doi:10.1016/j.pec.2008.08.003

8 Redeker C, Wardle J, Wilder D, *et al.* The launch of Cancer Research UK's "Reduce the Risk" campaign: baseline measurements of public awareness of cancer risk factors in 2004. *Eur J Cancer Oxf Engl 1990* 2009;**45**:827–36. doi:10.1016/j.ejca.2008.10.018

9 Cancer Research UK. Cancer Awareness Measure (CAM). Key Findings Report; 2014 & Trends Analysis (2008-2014). London, UK: 2016. http://www.cancerresearchuk.org/sites/default/files/cam_key_findings_report_-_2014_trends_analysis_v5.pdf

10 Marlow LAV, Robb KA, Simon AE, *et al.* Awareness of cancer risk factors among ethnic minority groups in England. *Public Health* 2012;**126**:702–9. doi:10.1016/j.puhe.2012.05.005

11 Wardle J, Waller J, Brunswick N, *et al.* Awareness of risk factors for cancer among British adults. *Public Health* 2001;**115**:173–4. doi:10.1038/sj/ph/1900752

12 Hawkins NA, Berkowitz Z, Peipins LA. What does the public know about preventing cancer? Results from the Health Information National Trends Survey (HINTS). *Health Educ Behav* 2010;**37**:490–503. doi:10.1177/1090198106296770

13 Stein K, Zhao L, Crammer C, *et al.* Prevalence and sociodemographic correlates of beliefs regarding cancer risks. *Cancer* 2007;**110**:1139–48. doi:10.1002/cncr.22880

14 Westhoff E, Maria de Oliveira-Neumayer J, Aben KK, *et al.* Low awareness of risk factors among bladder cancer survivors: new evidence and a literature overview. *Eur J Cancer* 2016;**60**:136–45. doi:10.1016/j.ejca.2016.03.071

For peer review only

15 Stewart DE, Cheung AM, Duff S, *et al.* Attributions of cause and recurrence in long-term breast cancer survivors. *Psychooncology* 2001;**10**:179–83.

16 Panjari M, Davis SR, Fradkin P, *et al.* Breast cancer survivors' beliefs about the causes of breast cancer. *Psychooncology* 2012;**21**:724–9. doi:10.1002/pon.1949

17 Petrie KJ, Myrtveit SM, Partridge AH, *et al.* The relationship between the belief in a genetic cause for breast cancer and bilateral mastectomy. *Health Psychol* 2015;**34**:473–6. doi:10.1037/hea0000118

18 Stubbings S, Robb KA, Waller J, *et al.* Development of a measurement tool to assess public awareness of cancer. *Br J Cancer* 2009;**101**:S13–S17.

19 Simon AE, Forbes LJL, Boniface D, *et al.* An international measure of awareness and beliefs about cancer: development and testing of the ABC. *BMJ Open* 2012;**2**:e001758. doi:10.1136/bmjopen-2012-001758

20 Smith SG, Osborne K, Tring S, *et al.* Evaluating the impact of a community-based cancer awareness roadshow on awareness, attitudes and behaviors. *Prev Med* 2016;**87**:138–43. doi:10.1016/j.ypmed.2016.02.034

21 McGrath R. *Twython*. 2013. Accessed online, https://twython.readthedocs.io/en/latest/#

22 Hsieh HF, Shannon SE. Three Approaches to Qualitative Content Analysis. *Qual Health Res* 2005;**15**:1277–88. doi:10.1177/1049732305276687

23 Pill J. The Delphi method: Substance, context, a critique and an annotated bibliography. *Socioecon Plann Sci* 1971;**5**:57–71. doi:10.1016/0038-0121(71)90041-3

24 DeVellis RF. *Scale Development: Theory and Applications*. Third edition. Thousand Oaks, Calif: SAGE Publications, Inc 2011.

25 Cohen P, Cohen J, Aiken LS, *et al.* The problem of units and the circumstance for POMP. *Multivar Behav Res* 1999;**34**:315–46. doi:10.1207/S15327906MBR3403_2

26 Rosseel Y. lavaan: An R Package for Structural Equation Modeling. *J Stat Softw* Published Online First: 2012. Accessed online, https://www.jstatsoft.org/article/view/v048i02

27 Wells G, Shea B, O'Connell D, Peterson J, Welch V, Losos M, Tugwell P. The Newcastle-Ottawa Scale (NOS) for assessing the quality of nonrandomised studies in meta-analyses. 2013, http://www.ohri.ca/programs/clinical_epidemiology/oxford.asp.

28 Shahab L, McGowan JA, Waller J, *et al.* Prevalence of beliefs about actual and mythical causes of cancer and their association with socio-demographic and health-related characteristics: Findings from a cross-sectional survey in England. *Eur J Cancer* 2018; doi:10.1016/j.ejca.2018.03.029

29 Robb KA, Stubbings S, Ramirez A, *et al.* Public awareness of cancer in Britain: a population-based survey of adults. *Br J Cancer* 2009;**101**:S18–23. doi:10.1038/sj.bjc.6605386

30 de Vocht F. Inferring the 1985–2014 impact of mobile phone use on selected brain cancer subtypes using Bayesian structural time series and synthetic controls. *Environ Int* 2016;**97**:100–7. doi:10.1016/j.envint.2016.10.019

For peer review only

31 Schüz J. Exposure to extremely low-frequency magnetic fields and the risk of childhood cancer: update of the epidemiological evidence. *Prog Biophys Mol Biol* 2011;**107**:339–42. doi:10.1016/j.pbiomolbio.2011.09.008

32 Saliev T, Begimbetova D, Masoud A-R, *et al.* Biological effects of non-ionizing electromagnetic fields: Two sides of a coin. *Prog Biophys Mol Biol* Published Online First: 17 July 2018. doi:10.1016/j.pbiomolbio.2018.07.009

33 Boxell EM, Smith SG, Morris M, *et al.* Increasing awareness of gynecological cancer symptoms and reducing barriers to medical help seeking: does health literacy play a role? *J Health Commun* 2012;**17 Suppl 3**:265–79. doi:10.1080/10810730.2012.712617

34 Power E, Simon A, Judzczyk D, *et al.* Assessing awareness of colorectal cancer symptoms: Measure development and results from a population survey in the UK. *BMC Cancer* 2011;**11**. doi:10.1186/1471-2407-11-366

35 Morse EP, Maegga B, Joseph G, *et al.* Breast Cancer Knowledge, Beliefs, and Screening Practices among Women Seeking Care at District Hospitals in Dar es Salaam, Tanzania. *Breast Cancer Basic Clin Res* 2014;**8**:73–9. doi:10.4137/BCBCR.S13745

36 Olanyinka A, Joel A, Feyisayo G. Knowledge and beliefs about breast cancer among female undergraduate in a selected private tertiary institution in Edo State Nigeria. *Int J Trop Med* 2012;**7**:151–6.

For peer review only

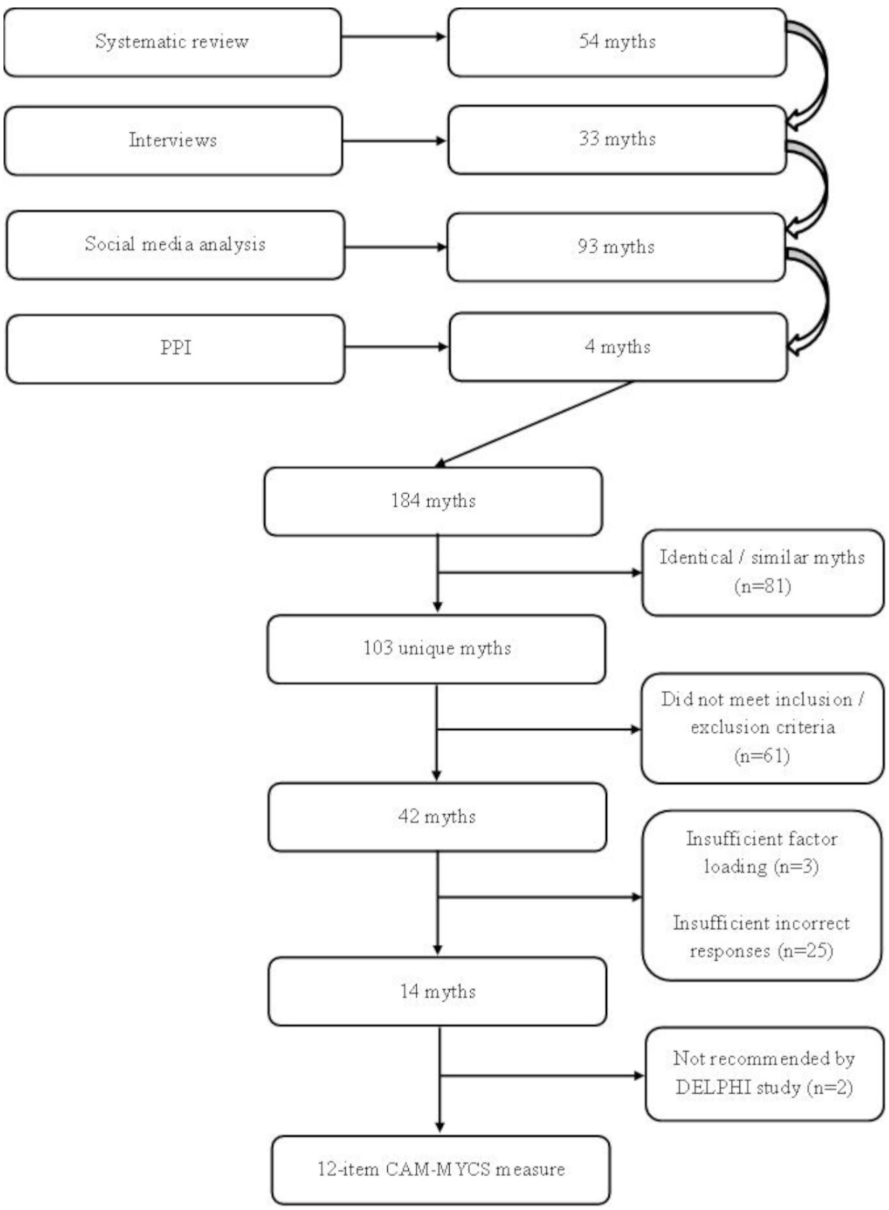

Flow chart of CAM-MYCS development

177x241mm (300 x 300 DPI)

Supplementary Table 1. Citation generated from search terms, May 2015

Step	Term Category	Search string	Medline/ EMBASE	PsycINFO/ EXTRA	All together-deduplicated
1	"Cancer" terms^	(cancer OR neoplasm).me	2,776,856	47,990	
2	"Belief" terms^	(caus* or attribute* or belie* or perce* or (illness adj representation*) or know* or aware* or thought* or idea*).me	697,570	1,386,598	
3	"Wrong" terms	(false* or incorrect* or myth* or erroneous* or wrong* or mistaken* or error or untrue or fallacious).mp	921,613	125,798	
4	"Cancer patient" terms	cancer adj patient*.tw	302,437	11,606	
5	Combination	1 AND 2 AND 3	784	534	
6	Exclusion	5 NOT 4	716	460	
7	Limits	Human, English	632	417	**999**

^Mesh terms/Subject heading terms for Medline/Embase only (otherwise .tw)

Supplementary information

Appendix A: Topic guide

SEMI-STRUCTURED INTERVIEW SCHEDULE (CANCER AWARENESS)

Objective
In-depth exploration of the incorrect causal beliefs about cancer in the UK general population sample and identifying existing socioeconomic inequalities.

To generate items to develop a valid and reliable tool for assessing cancer awareness in the public.

For peer review only

Beginning the interview

- Introduction of researcher; topics to be covered/structure of interview; confidentiality; timing; confirming consent for tape-recording

1.1 Background

- Name RP, a masters' student in Health Psychology at UCL
- Interested in understanding and knowing about people's understanding of cancer. Don't worry there are no right or wrong answers. We are just interested in your ideas.

1.2 Interview Outline

- If that's ok, I will ask you a couple of questions about your views on cancer in general,
- Also interested to hear about your beliefs about the different causes of cancer
- At the end will describe a new cancer awareness assessment tool we are developing at UCL and I would very much appreciate to hear your views about that tool

1.3 Interview Arrangements

- Should take around half an hour, but we might find lots to talk about, so please go into as much detail as possible.
- Ok to record interview (consent)
- Of course it's all completely anonymous/confidential and recordings will be transcribed

During the interview (main themes to be covered)

2.1. Questions exploring the causal beliefs or risk perceptions of cancer:

2.1.1. How much do you understand about cancer? Have you have had any experience with cancer through friends or relatives?

2.1.2. A number of different things have been linked to the development of cancer, but new discoveries are often being made. What do you think are the most common causes of cancer?

2.1.3. What things do you think affects a person's chances of developing cancer?

2.1.4. What are the best ways to avoid getting cancer?

2.1.5. Do you think that certain behaviours or risk factors are more important for specific people (e.g. prompt: men vs. women, younger vs. older; different ethnic groups)?

2.1.6 We have been asking all our interviewees this question, what do you think our chances of developing cancer is in your lifetime? Would you say it is higher or lower compared to an average person?

Prompts for questions:

- "What else?"
- "Take your time to think about it"

3. Ending the interview

- Anything else participants would like to say?
- Any important issues not raised?
- Any other questions regarding research?
- Debriefing about study
- Provision of researcher (RP) and principal researcher's (LS/SS) contact information, should anything else come up
- Thanks

For peer review only

LONDON'S GLOBAL UNIVERSITY

UCL

Cancer Information Leaflet

Known risk factors and common myths

IBAC Study Educational Leaflet

By: Emma Fox

What is cancer?

Cancer is the name of a collection of diseases where the cells of the body begin to divide abnormally. These cells can become invasive, and grow out of control, spreading into surrounding tissues. Many cancers grow to form solid masses of tissue, known as tumours.

Cancer can start almost anywhere in the human body, and there are more than 100 different types of cancer.

What causes cancer?

Changes to genes, called mutations, play a vital role in the development of cancer. Some of the gene changes that lead to cancer may be inherited. Most gene mutations, however, happen after birth.

It is not usually possible to say exactly how a person develops cancer. Nevertheless, research has shown that certain risk factors may increase the likelihood of an individual developing cancer. These include factors such as exposure to ultraviolet rays found in sunlight, as well as lifestyle behaviours such as smoking and heavy alcohol consumption. Other risk factors include family history and age.

Myths and misconceptions about cancer

In recent years, information regarding cancer prevention and treatment has improved. However, some of the information that is spread can be misleading or inaccurate. Becoming more aware of correct known risk factors as well as incorrect cancer myths may allow individuals to make more informed decisions regarding their health.

For peer review only

Cancer prevention- myths and facts

The list below expands on some of the most-researched known risk factors for cancer. It will also address common misconceptions and questions regarding each risk factor.

Smoking

People who use tobacco products or are regularly around environmental smoke (otherwise known as second-hand smoke) have an increased risk of developing cancer. This is because tobacco products have many chemicals inside them that directly damage DNA.

Is there use in quitting if a person has been smoking for a long time?

Yes. The damage done by smoking is cumulative, meaning that the longer an individual smokes, the higher their risk of developing cancer becomes. However, quitting at any age brings immediate benefits. Ten years after quitting, former smokers cut their risk of developing lung cancer by up to 50%.

Alcohol

Multiple research studies have found an association between heavy alcohol consumption and cancer. The more alcohol a person drinks regularly over time, the higher their risk of developing cancer will be.

Can drinking red wine help prevent cancer?

Most likely, no. Research studies have found that certain properties of red wine, such as resveratrol, may have anticancer properties. However, subsequent trials on humans have not shown any convincing evidence that these properties are effective in preventing or treating cancer.

Radiation

Radiation of particular wavelengths, called ionizing radiation, has enough energy to directly damage DNA and lead to cancer. This includes forms of high-energy radiation such as radon, ex-rays, and gamma rays.

Do cell phones and microwaves cause cancer?

No. Lower-energy, non-ionizing forms of radiation, such as the light and energy given off from cell phones, power lines, and microwaves does not damage DNA and has not been found to cause cancer.

Sunlight

The sun, tanning booths, and sun beds all give off ultraviolet (UV) radiation. Exposure to these UV rays can cause skin damage and increase the risk of getting skin cancer.

Are people with naturally olive or dark skin at risk of getting skin cancer?

Yes. Although people with lighter skin have an increased risk of getting skin cancer, anyone can burn if the sun is strong enough. The darker an individual's skin, the more melanin pigment they have, which protects the skin from UV rays, reducing their cancer risk. However, this does not mean that people with darker skin cannot get skin cancer. Often, people with darker skin will develop skin cancer on parts of their body that aren't usually exposed to the sun, such as on their palms or on the soles of their

Diet

Maintaining a healthy body weight and eating a well-balanced and healthy diet can reduce the risk of many cancers. Research has shown that regular consumption of fruits, vegetables, and high fibre foods can prevent the onset of cancer. In addition, frequent consumption of red and processed meats and salt can increase the risk of developing cancer.

Do artificial sweeteners cause cancer?

No. There are many stories in the media about various foods that are claimed to increase or decrease the risk of cancer. Some of these associations, however, do not have enough scientific evidence to support them.

For example, there is no evidence that the artificial sweeteners that are currently in the market are related to cancer risk in humans. Questions relating to artificial sweeteners and cancer risk came out in the 1980s when a study found a link between cyclamate (a type of artificial sweetener) and bladder cancer in laboratory rats. However, since that time, a large body of research strongly suggests that artificial sweeteners do not cause cancer in

For peer review only

STROBE 2007 (v4) Statement—Checklist of items that should be included in reports of *cross-sectional studies*

Section/Topic	Item #	Recommendation	Reported on page #
**Title and abstract**	1	(*a*) Indicate the study's design with a commonly used term in the title or the abstract	1
		(*b*) Provide in the abstract an informative and balanced summary of what was done and what was found	2
**Introduction**			
Background/rationale	2	Explain the scientific background and rationale for the investigation being reported	4-5
Objectives	3	State specific objectives, including any prespecified hypotheses	4-5
**Methods**			
Study design	4	Present key elements of study design early in the paper	6-10
Setting	5	Describe the setting, locations, and relevant dates, including periods of recruitment, exposure, follow-up, and data collection	6-10
Participants	6	(*a*) Give the eligibility criteria, and the sources and methods of selection of participants	6-10
Variables	7	Clearly define all outcomes, exposures, predictors, potential confounders, and effect modifiers. Give diagnostic criteria, if applicable	6-10
Data sources/ measurement	8*	For each variable of interest, give sources of data and details of methods of assessment (measurement). Describe comparability of assessment methods if there is more than one group	6-10
Bias	9	Describe any efforts to address potential sources of bias	*10-11*
Study size	10	Explain how the study size was arrived at	6-10
Quantitative variables	11	Explain how quantitative variables were handled in the analyses. If applicable, describe which groupings were chosen and why	9-11
Statistical methods	12	(*a*) Describe all statistical methods, including those used to control for confounding	9-11
		(*b*) Describe any methods used to examine subgroups and interactions	9-11
		(*c*) Explain how missing data were addressed	9-11
		(*d*) If applicable, describe analytical methods taking account of sampling strategy	9-11
		(*e*) Describe any sensitivity analyses	9-11
**Results**			

For peer review only

Participants	13*	(a) Report numbers of individuals at each stage of study—eg numbers potentially eligible, examined for eligibility, confirmed eligible, included in the study, completing follow-up, and analysed	12-14
		(b) Give reasons for non-participation at each stage	12-14
		(c) Consider use of a flow diagram	Figure
Descriptive data	14*	(a) Give characteristics of study participants (eg demographic, clinical, social) and information on exposures and potential confounders	12 (tables)
		(b) Indicate number of participants with missing data for each variable of interest	12 (tables)
Outcome data	15*	Report numbers of outcome events or summary measures	12-14 (tables)
Main results	16	(*a*) Give unadjusted estimates and, if applicable, confounder-adjusted estimates and their precision (eg, 95% confidence interval). Make clear which confounders were adjusted for and why they were included	12-14
		(*b*) Report category boundaries when continuous variables were categorized	6-10
		(*c*) If relevant, consider translating estimates of relative risk into absolute risk for a meaningful time period	N/A
Other analyses	17	Report other analyses done—eg analyses of subgroups and interactions, and sensitivity analyses	N/A
**Discussion**			
Key results	18	Summarise key results with reference to study objectives	15-16
Limitations	19	Discuss limitations of the study, taking into account sources of potential bias or imprecision. Discuss both direction and magnitude of any potential bias	15-16
Interpretation	20	Give a cautious overall interpretation of results considering objectives, limitations, multiplicity of analyses, results from similar studies, and other relevant evidence	15-16
Generalisability	21	Discuss the generalisability (external validity) of the study results	15-16
**Other information**			
Funding	22	Give the source of funding and the role of the funders for the present study and, if applicable, for the original study on which the present article is based	17

*Give information separately for cases and controls in case-control studies and, if applicable, for exposed and unexposed groups in cohort and cross-sectional studies.

Note: An Explanation and Elaboration article discusses each checklist item and gives methodological background and published examples of transparent reporting. The STROBE checklist is best used in conjunction with this article (freely available on the Web sites of PLoS Medicine at http://www.plosmedicine.org/, Annals of Internal Medicine at http://www.annals.org/, and Epidemiology at http://www.epidem.com/). Information on the STROBE Initiative is available at www.strobe-statement.org.

For peer review only
